# The cytokines interleukin-6 and interferon-α induce distinct microglia phenotypes

**DOI:** 10.1186/s12974-022-02441-x

**Published:** 2022-04-16

**Authors:** Phillip K. West, Andrew N. McCorkindale, Boris Guennewig, Thomas M. Ashhurst, Barney Viengkhou, Emina Hayashida, So Ri Jung, Oleg Butovsky, Iain L. Campbell, Markus J. Hofer

**Affiliations:** 1 School of Life and Environmental Sciences, The University of Sydney, Charles Perkins Centre and the Sydney Institute for Infectious Diseases, Sydney, NSW Australia; 2 Discipline of Pathology, School of Medical Sciences, Faculty of Medicine and Health, Charles Perkins Centre, The University of Sydney, Sydney, NSW Australia; 3 Sydney Medical School, Brain and Mind Centre, The University of Sydney, Sydney, NSW Australia; 4 Sydney Cytometry Core Facility, The University of Sydney and Centenary Institute, Sydney, NSW Australia; 5 Center for Neurologic Diseases, Department of Neurology, Brigham and Women’s Hospital, Harvard Medical School, Boston, MA USA

**Keywords:** Microglia, Interleukin-6, Interferon-alpha, Cytokine, Phenotype, Central nervous system, Neuroinflammation

## Abstract

**Background:**

Elevated production of the cytokines interleukin (IL)-6 or interferon (IFN)-α in the central nervous system (CNS) is implicated in the pathogenesis of neurological diseases such as neuromyelitis optica spectrum disorders or cerebral interferonopathies, respectively. Transgenic mice with CNS-targeted chronic production of IL-6 (GFAP-IL6) or IFN-α (GFAP-IFN) recapitulate important clinical and pathological features of these human diseases. The activation of microglia is a prominent manifestation found both in the human diseases and in the transgenic mice, yet little is known about how this contributes to disease pathology.

**Methods:**

Here, we used a combination of ex vivo and in situ techniques to characterize the molecular, cellular and transcriptomic phenotypes of microglia in GFAP-IL6 versus GFAP-IFN mice. In addition, a transcriptomic meta-analysis was performed to compare the microglia response from GFAP-IL6 and GFAP-IFN mice to the response of microglia in a range of neurodegenerative and neuroinflammatory disorders.

**Results:**

We demonstrated that microglia show stimulus-specific responses to IL-6 versus IFN-α in the brain resulting in unique and extensive molecular and cellular adaptations. In GFAP-IL6 mice, microglia proliferated, had shortened, less branched processes and elicited transcriptomic and molecular changes associated with phagocytosis and lipid processing. In comparison, microglia in the brain of GFAP-IFN mice exhibited increased proliferation and apoptosis, had larger, hyper-ramified processes and showed transcriptomic and surface marker changes associated with antigen presentation and antiviral response. Further, a transcriptomic meta-analysis revealed that IL-6 and IFN-α both contribute to the formation of a core microglia response in animal models of neurodegenerative and neuroinflammatory disorders, such as Alzheimer’s disease, tauopathy, multiple sclerosis and lipopolysaccharide-induced endotoxemia.

**Conclusions:**

Our findings demonstrate that microglia responses to IL-6 and IFN-α are highly stimulus-specific, wide-ranging and give rise to divergent phenotypes that modulate microglia responses in neuroinflammatory and neurodegenerative diseases.

**Supplementary Information:**

The online version contains supplementary material available at 10.1186/s12974-022-02441-x.

## Background

Interleukin (IL)-6 and interferon (IFN)-α are cytokines that have essential roles in regulating inflammatory processes in the central nervous system (CNS) [1]. The chronic production of these cytokines is directly involved in the pathogenesis of several neuroinflammatory and neurodegenerative diseases, such as neuromyelitis optica spectrum disorder (NMOSD) and cerebral interferonopathies, respectively [1, 2]. For example, in the case of NMOSD, antibody-mediated neutralization of the IL-6 receptor ameliorates disease [3] while inhibition of the IFN-α signaling molecule JAK1 has shown promising outcomes in patients with cerebral interferonopathies [4]. These diseases are debilitating, there is no cure and additional research is required to clarify the underlying pathology to increase the efficacy and precision of new therapeutics [3, 5, 6].

Important clinical and pathological phenotypes of these diseases are recapitulated in transgenic mouse models with CNS-restricted, astrocyte-targeted production of IL-6 or IFN-α, termed glial fibrillary acidic protein (GFAP)-IL6 or GFAP-IFN mice, respectively [1, 7, 8]. GFAP-IL6 mice exhibit signs of disease such as impaired hippocampal long-term potentiation [9, 10], neuronal hyperexcitability [10, 11], progressive cognitive decline [12] and development of ataxia [2, 8]. Pathological changes in the CNS of GFAP-IL6 mice include neurodegeneration and demyelination [12–14], reactive astrogliosis and microgliosis [8, 12, 14], upregulation of acute-phase response proteins [8, 15, 16], proliferative angiopathy [8] and breakdown of blood–brain barrier integrity [13, 17]. This is similar to patients with NMOSD, who have increased intrathecal levels of IL-6 [18–21], reactive astrogliosis and microgliosis and destructive demyelination [22–25] and can clinically present with ataxia and seizures [26, 27]. On the other hand, GFAP-IFN mice exhibit a cerebral interferonopathy characterized by stunted growth, neuronal hyperexcitability, cognitive dysfunction, ataxia, convulsive seizures and increased mortality [28]. In the CNS, these animals show reactive astrogliosis and microgliosis, neurodegeneration, microangiopathy with aneurysms and cerebral calcification in the cerebellum and thalamus [7, 28, 29], features also commonly seen in patients with cerebral interferonopathies such as Aicardi–Goutières syndrome [30–33].

Microglia are direct responders to both IL-6 and IFN-α and the increased activation of these cells is a common feature of both IL-6- and IFN-α-mediated diseases in humans and the cytokine-transgenic mice [2]. Yet, little is known about how this feature contributes to these diverse neuropathologies. Microglia are the primary immune cells of the CNS and are highly plastic, with remarkable abilities to fine-tune their molecular and functional phenotype to different states depending on the nature, duration and context of the stimulus. In line with this ability, these cells can exist in a vast spectrum of molecular and functional phenotypes [34–38]. Thus, we hypothesized that microglia are both a prominent target and effector cell of IL-6 and IFN-α in the CNS. We investigated how IL-6 and IFN-α alter the molecular and cellular phenotype of microglia in the GFAP-IL6 versus GFAP-IFN mice. We also correlated the unique phenotypes of microglia in the mouse models to their transcriptomic responses. Finally, we performed a transcriptomic meta-analysis and determined that microglia from other neuropathological states exhibit IL-6 or IFN-α-like responses.

## Methods

### Mice

The transgenic MacGreen, GFAP-IL6 and GFAP-IFN mice were described previously [7, 8, 28, 39] and were bred and maintained under specific-pathogen-free conditions at the animal facility of the University of Sydney. Both GFAP-IL6 and GFAP-IFN mice were originally developed by I.L. Campbell at the Scripps Research Institute, La Jolla, CA, USA and breeding stock were obtained from there. For the RNA-seq and flow cytometry experiments, GFAP-IL6 and GFAP-IFN mice were crossed with MacGreen mice, which encode the enhanced green fluorescent protein (eGFP) gene under the control of the *Csf1r* promoter, labeling the myeloid cell compartment, including microglia and macrophages, with eGFP [39]. MacGreen and GFAP-IL6 mice were on C57BL/6 background and GFAP-IFN mice were on a mixed C57BL/6 × BALB/c background. Wildtype littermates from both GFAP-IL6 and GFAP-IFN lines were used as WT controls and no differences were found between lines. The C57BL/6 WT controls for RNA-seq were validated by performing RTPCR for key microglia genes in WT controls from both lines. Animals received food and water ad libitum. The temperature and humidity parameters in animal holding areas were set to fall between 20–24 °C and 40–70%, respectively, with light between 0545 and 1745 h. Mice were housed at a maximum density of 6 mice per cage.

For the microglia cell turnover experiment, 1-, 3- and 6-month-old mice were injected intraperitoneally with 100 mg/kg 5-bromo-2’-deoxyuridine (BrdU) in 0.9% (w/v) NaCl each day at 1500 h for 5 days to label proliferating cells. For histological analysis, mice were deeply anaesthetized with isoflurane, perfused intracardially with ice-cold phosphate-buffered saline (PBS) followed by ice-cold, neutral buffered 4% paraformaldehyde (PFA). Brains were collected, fixed in 4% neutral buffered PFA overnight at 4 °C and then paraffin-embedded. For passive tissue clearing, PFA-fixed brains were dissected into the cerebellum and cortex and were transferred to PBS. For Western blot, 1-month-old mice were euthanized with isoflurane and the cerebellum was dissected and flash frozen. For ex vivo experiments, mice were deeply anaesthetized with isoflurane, perfused intracardially with ice-cold PBS and the brain was isolated.

### Histology

For histochemistry/immunohistochemistry (HC/IHC), paraffin sections (12 μm) were deparaffinized and rehydrated in graded ethanol. Antigens were unmasked with 25 mM Tris pH 8, 5 mM EDTA pH 8 and 0.05% (w/v) SDS in a vegetable steamer for 40 min. Sections were incubated in 0.3% peroxidase for 10 min and blocked in 1% goat serum with 0.1% Triton X-100 and 0.05% Tween-20 in PBS for 30 min. The primary antibodies rabbit anti-pY701-STAT1 (sc135648, Santa Cruz Biotechnology, 1:100) or rabbit anti-pY705-STAT3 (9145L, Cell Signaling Technologies, 1:100) were incubated overnight at 4 °C. Following primary antibody incubation and washing, sections were incubated with biotinylated secondary goat anti-rabbit antibodies (BA-1000, Vector Laboratories, 1:200) followed by VECTASTAIN Elite ABC Horseradish Peroxidase (HRP) Kit (PK-7200, Vector Laboratories) for 1 h at room temperature (RT). Sections were developed with 3,3’-diaminobenzidine with nickel enhancement (SK-4100, Vector Laboratories). Following washing, avidin/biotin blocking (SP-2001, Vector Laboratories) was performed. Biotinylated-tomato lectin (L0651, Sigma-Aldrich, 1:50) was incubated overnight at 4 °C. Sections were then washed and incubated with HRP-conjugated streptavidin (SA-5004, Vector Laboratories, 1:200) for 1 h at RT. Sections were developed with 3,3′-diaminobenzidine without nickel enhancement, counterstained with Mayer’s hematoxylin and mounted. Sections were viewed with a DM4000B microscope (Leica Microsystems) and imaged using a SPOT Flex 15.2 64 Mp Shifting Pixel camera and SPOT Advanced 4.5 software (Diagnostic Instruments).

For immunofluorescence, deparaffinization, rehydration, antigen retrieval and blocking were performed as described above. The primary antibodies rabbit anti-Iba1 (019–19741, Wako Pure Chemical Industries, 1:500) and rat anti-BrdU (MCA2060GA, BioRad Laboratories, 1:100) were incubated overnight at 4 °C. Following primary antibody incubation and washing, sections were incubated with secondary goat anti-rabbit IgG-AF488 (A-11034, Thermo Fisher Scientific, 1:500) and goat anti-rat IgG-AF594 (A-11007, Thermo Fisher Scientific, 1:500) for 1 h at RT. Slides were washed and then cover-slipped with Fluoroshield™ DAPI mounting media (Sigma-Aldrich). For the TUNEL assay, the primary antibody rabbit anti-Iba1 (1:500) was incubated for 2 h at RT. Following washing, sections were incubated in TUNEL reaction mixture (TMR Red, Roche) and secondary goat anti-rabbit IgG-AF488 (1:500) for 1 h at 37 °C. Slides were washed and then cover-slipped with Fluoroshield™ DAPI mounting media (Sigma-Aldrich). Fluorescent imaging was performed at the Advanced Microscopy Facility of the Bosch Institute at the University of Sydney using a Zeiss AxioScan.Z1 slide-scanning microscope at 20 × magnification and a Zeiss LSM800 confocal laser scanning microscope using 20 × Plan Apochromat NA = 0.8 air objective, 40 × Plan Apochromat NA = 1.3 oil-immersion objective, or 63 × Plan Apochromat NA = 1.4 oil-immersion objective with 405, 488 and 561 nm lasers and appropriate filters (Carl Zeiss).

### Immunoblotting

Total protein for immunoblotting was isolated from the cerebella of mice in 100 mM Tris pH 7.5, 150 mM NaCl, 1 mM EDTA, 1% deoxycholic acid, 1% Triton X-100, 0.1% (w/v) SDS, 2 mM PMSF, 50 mM NaF, and 1 × Protease Inhibitor Cocktail III (539134, Merck Millipore) and 1 × Phosphatase Inhibitor Cocktail II (524625, Merck Millipore). Proteins were separated on a 10% Tris–glycine polyacrylamide gel by electrophoresis and transferred onto polyvinylidene fluoride membranes. The primary antibodies rabbit anti-pY701-STAT1 (7649, Cell Signaling Technology, 1:2000), rabbit anti-pS727-STAT1 (9177, Cell Signaling Technology, 1:2000), rabbit anti-pY705-STAT3 (9131, Cell Signaling Technology, 1:2000), rabbit anti-STAT1 (9172, Cell Signaling Technology, 1:2000), rabbit anti-STAT3 (4904, Cell Signaling Technology, 1:2000) and mouse anti-GAPDH (MAB374, Merck Millipore, 1:30,000) in 5% (w/v) skim milk powder in Tris-buffered saline and 0.1% Tween-20 were incubated with membranes overnight at 4 °C. Membranes were incubated with HRP-conjugated goat anti-rabbit IgG (7074P2, Cell Signaling Technology, 1:10,000) and peroxidase-conjugated goat anti-mouse IgG (A0168, Sigma-Aldrich, 1:10,000) for 1 h at RT. Proteins were detected by chemiluminescence (WBKLS0500, Merck Millipore) and visualized using an iBright™ 1500 (Thermo Fisher Scientific). Relative protein bands were quantified by densitometry using ImageJ software (NIH, USA) by normalizing band density to that of the corresponding GAPDH loading control.

### Tissue clearing and microglia morphological analysis

PFA-fixed cerebella and cortices were incubated in 4% (w/v) acrylamide, 0.05% (w/v) bisacrylamide and 0.25% (w/v) VA-044 (Wako Pure Chemical Industries) in PBS for 48 h at 4 °C and were then incubated at 37 °C for 3 h to polymerize the hydrogel-tissue matrix. Polymerized tissues were next briefly washed, transferred into 8% (w/v) SDS in PBS and incubated at 37 °C with shaking at 225 rpm until tissues became transparent (4–5 days). Cleared tissues were then washed in PBS for 6 h at RT, with fresh changes of PBS each hour. Rabbit anti-Iba1 (019–19741, Wako Pure Chemical Industries, 1:200) in 2% goat serum with 0.1% Triton X-100 and 0.01% sodium azide in PBS was incubated with tissues at RT with shaking for 7 days. Following 6 h of washing, tissues were then incubated with secondary goat anti-rabbit IgG-AF594 (A-11037, Thermo Fisher Scientific, 1:200) at RT with shaking for 7 days. Following washing, tissues were incubated overnight in 88% (w/v) Histodenz (Sigma-Aldrich), 0.01% sodium azide and 1 μg/mL Hoechst 33342 (B2261, Sigma-Aldrich) in PBS at RT with shaking. Imaging was performed on a Zeiss LSM800 confocal laser scanning microscope using 20 × Plan Apochromat NA = 0.8 air objective with 405 and 568 nm lasers and appropriate filters (Carl Zeiss). Three-dimensional reconstructions of microglia were generated using the Filament Tracer feature in Imaris 9.2.1 (Oxford Instruments). Following surface and dendrite rendering, morphometric analysis was performed using the in-house statistics generator. The total process length, total process volume, number of branching points, number of terminal points, and total Sholl intersections were analyzed as indicators of microglia process size and complexity.

### Fluorescence-activated cell sorting (FACS) of microglia

PBS-perfused brains were washed in PBS, the cerebellum was dissected and cells were mechanically dissociated by Dounce homogenization and passed through a 70 μm sieve. Cells were pelleted by centrifugation at 460*g* for 10 min at 4 °C before resuspension in 70% (v/v) Percoll. An equal volume of 37% (v/v) Percoll was slowly layered on top of the cell suspension to form a discontinuous Percoll gradient. Microglia and leukocytes were collected from the 37/70% (v/v) interface after centrifugation at 1825*g* for 25 min at 25 °C with no brake. Cells were then stained on ice with rat anti-4D4 (clone 4D4, 1:300 [40], provided by Oleg Butovsky) in 5% FBS (Thermo Fisher Scientific) and 5 mM EDTA in PBS for 25 min. Following washing, cells were then incubated on ice with goat anti-rat IgG-APC (Poly4054, Biolegend, 1:300). Cells were washed and then incubated on ice with 7AAD (51-68981E, BD Bioscience, 1:20) for 10 min. Using a BD Influx™ Cell Sorter (BD Bioscience), eGFP^+^ 4D4^+^ 7AAD^–^ cerebellar microglial cells were sorted into lysis buffer. Total RNA was isolated from FACS-sorted microglia using the RNeasy® plus micro kit (QIAGEN Inc.) according to the manufacturer’s instructions.

### Library preparation for RNA-seq

The cDNA was synthesized from cerebellar microglial cell RNA at the Western Sydney University Next Generation Sequencing facility using SMARTer® Stranded Total RNA-Seq Kit—Pico Input Mammalian (Takara Bio Inc.) according to manufacturer’s instructions. The RNA-seq library fragments were amplified by PCR, using 14 enrichment cycles, before purification and RNA-seq.

### RNA-seq

Paired-end, 126 bp read length RNA-seq was performed on a HiSeq 2500 (Illumina) at the Western Sydney University Next Generation Sequencing facility.

### RNA-seq analysis

RNA-seq data were processed using a custom pipeline designed by Boris Guennewig [41–43]. The quality of the input data was assessed using FastQC (version 0.11.3) [44] and reads were mapped to the mm10 reference genome using the STAR aligner (version 2.5.2a) [45]. Potential transcripts were identified using StringTie (version 1.3.3b) [46] and known GENCODE genes were quantified using RSEM (version 1.3.0) [47]. For differential expression analysis, RSEM count data were imported into the R project environment [48]. Reads with primarily zero counts were filtered out to obtain the genes for downstream analyses. Outlier samples were identified using principal component analysis (PCA) and hierarchical clustering. Reads were normalized using the trimmed mean of means (TMM) and differential expression analysis was performed in R using the edgeR package [49]. A differentially expressed gene (DEG) was called significant if its false discovery rate (FDR) was ≤ 0.05 after Benjamini–Hochberg correction. Significantly enriched biological processes were identified using the gene ontology (GO) enrichment tool WebGestalt [50] and clustering of biological processes was visualized using Enrichment Map [51]. Two-way analysis was performed using the DEG lists generated by edgeR. Genes that were at least twofold differentially expressed by GFAP-IL6 versus WT microglia and/or genes at least twofold differentially expressed by GFAP-IFN versus WT microglia were plotted. A constant of 0.125 was added to the normalized reads prior to the fold-change calculation to avoid infinite fold-change values for genes with zero counts across a group. “Core response” genes were differentially expressed in both GFAP-IL6 versus WT microglia and GFAP-IFN versus WT microglia, with no significant difference in expression between GFAP-IL6 versus GFAP-IFN microglia. “IL-6-skewed” genes were differentially expressed in GFAP-IL6 versus WT microglia, but not GFAP-IFN versus WT microglia, or were differentially expressed by both GFAP-IL6 versus WT and GFAP-IFN versus WT microglia but were differentially expressed by GFAP-IL6 versus GFAP-IFN microglia, or were genes that were significantly upregulated by GFAP-IL6 versus WT microglia and significantly downregulated by GFAP-IFN versus WT microglia. “IFN-α-skewed” genes were differentially expressed in GFAP-IFN versus WT microglia, but not GFAP-IL6 versus WT microglia, or were differentially expressed by both GFAP-IFN versus WT and GFAP-IL6 versus WT microglia but were also differentially expressed by GFAP-IFN versus GFAP-IL6 microglia, or were genes that were significantly upregulated by GFAP-IFN versus WT microglia and significantly downregulated by GFAP-IL6 versus WT microglia. Significantly enriched biological processes were identified and visualized as above.

### RNA-seq meta-analysis

In addition to our RNA-seq data, RNA-seq FASTQ files were downloaded from the sequence read archive (SRA) and processed using the custom pipeline designed by Boris Guennewig. The files downloaded from SRA included WT Clec7a^–^, APP-PS1 Clec7a^–^ and APP-PS1 Clec7a^+^ microglia (GSE102563, n = 6 per group) [52]; non-transgenic (Non-Tg) and hMAPT-P301S microglia (GSE93180, n = 6 per group) [53]; unmanipulated, EAE CD11c^–^ and EAE CD11c^+^ microglia (GSE78809, n = 3 per group) [54]; and vehicle and LPS microglia (GSE75246, n = 5 per group) [55]. Data processing and DEG analysis was performed as described above and was performed separately for each study. Normalized gene reads were then Z-score transformed within each study before the data from the different studies were combined. Z-score transformation has been used previously to account for the “batch” effect of combining data from different studies [53]. The pheatmap package was used in R [56] to perform clustering analysis and generate the heatmap. For each gene cluster, significantly enriched biological processes were identified using WebGestalt [50]. For the genes in each cluster, we calculated log_2_-fold changes by comparing each sample group with its respective control, as well as the median log_2_-fold change for each comparison. A constant of 0.125 was added to the normalized reads prior to the fold-change calculation to avoid infinite fold-change values for genes with zero counts across a group.

### Microglia cDNA synthesis and real-time PCR (RTPCR) analysis

Following FACS-sorting of microglia from the whole brain and RNA isolation as above, cDNA was synthesized using RevertAid First Strand cDNA synthesis kit (Thermo Fisher Scientific) according to the manufacturer’s instructions. For each sample, we used cDNA from the same number of cells to normalize the RTPCR. Accordingly, the cDNA was diluted such that RTPCR was performed on cDNA from 250 microglial cells. The RTPCR was set up using SensiFAST™ SYBR® Lo-ROX (Meridian Bioscience) and 400 nM primer pairs and was performed with a 7500 Fast Real-Time PCR System (Thermo Fisher Scientific) using the ΔΔCt setting with the cycle program: 95 °C for 2 min, then 40 cycles of 95 °C for 5 s then 60 °C for 30 s, followed by melt curve analysis. The C_T_ for each gene of interest was normalized to the C_T_ of the housekeeping gene 18S rRNA. Primer sequences: *Apoe* (forward: TGTGGGCCGTGCTGTTGGTC; reverse: GCCTGCTCCCAGGGTTGGTTG) [57], *Axl* (forward: TGAGCCAACCGTGGAAAGAG; reverse: AGGCCACCTTATGCCGATCTA) [58], *B2m* (forward: GTGACCCTGGTCTTTCTGGT; reverse: GTATGTTCGGCTTCCCATTC) [59], *C4b* (forward: GACAAGGCACCTTCAGAACC; reverse: CAGCAGCTTAGTCAGGGTTACA), *Cst3* (forward: CGCTCCTTGCTGTTCCTGCT; reverse: TGCCCTTGTTGTACTCGCTCAC) [60], *Ctsb* (forward: AGACCTGCTTACTTGCTGTG; reverse: GGAGGGATGGTGTATGGTAAG) [61], *Fn1* (forward: ACCGACAGTGGTGTGGTCTA; reverse: CACCATAAGTCTGGGTCACG) [62], H2-D1 (forward: TCCGAGATTGTAAAGCGTGAAGA; reverse: GAACCCAAGCTCACAGGGAA) [63], *Slc39a14* (forward: GGAACCCTCTACTCCAACGC; reverse: ATGGTTATGCCCGTGATGGT) [64] and *18S* (forward: CACGGCCGGTACAGTGAAAC; reverse: AGAGGAGCGAGCGACCAA) [65].

### Flow cytometry

Following ex vivo isolation, single-cell suspensions of microglia and leukocytes from the brain were incubated at 4 °C for 30 min with rat anti-CD16/32-BB700 (2.4G2, BD Bioscience, 1:200) and LIVE/DEAD™ Fixable Blue Dead Cell Stain (Thermo Fisher Scientific, 1:500). Cells were washed and then stained at 4 °C for 30 min with fluorophore-conjugated antibodies: rat anti-B220-BUV661 (RA3-6B2, BD Bioscience, 1:200), rat anti-CD115-AF594 (AFS98, Biolegend, 1:200), rat anti-CD11b-BUV737 (M1/70, BD Bioscience, 1:200), Armenian hamster anti-CD11c-APC-R700 (N418, BD Bioscience, 1:200), hamster anti-CD3e-PE/Cy5.5 (145-2C11, Thermo Fisher Scientific, 1:200), rat anti-CD4-BV570 (RM4-5, Biolegend, 1:200), rat anti-CD45-APC/Cy7 (30-F11, BD Bioscience, 1:200), rat anti-CD62L-BV650 (MEL-14, Biolegend, 1:200), mouse anti-CD64-APC (X54-5/7.1, BD Bioscience, 1:200), rat anti-CD69-BV785 (H1.2F3, BD Bioscience, 1:200), hamster anti-CD80-PE/CF594 (16-10A1, BD Bioscience, 1:200), rat anti-CD86-BV605 (GL1, Biolegend, 1:200), rat anti-CD8a-BUV805 (53–6.7, BD Bioscience, 1:200), rat anti-F4/80-BUV395 (T45-2342, BD Bioscience, 1:200), rat anti-Ly6C-PE/Cy7 (HK1.4, Biolegend, 1:200), rat anti-Ly6G-BUV563 (1A8, BD Bioscience, 1:200), rat anti-MHC-II (I-A/I-E)-BV510 (M5/114.15.2, BD Bioscience, 1:200), mouse anti-NK1.1-PE/Cy5 (PK136, Biolegend, 1:200), rat anti-SCA-1-BV711 (D7, Biolegend, 1:200) and rabbit anti-TMEM119-PE (106–6, Abcam, 1:200). Cells were then fixed in 4% PFA prior to analysis performed on an LSR-X flow cytometer (BD Bioscience) and data were analyzed with FlowJo software version 10 (BD Bioscience).

Alternatively, cells were incubated at 4 °C for 30 min with unconjugated rat anti-CD16/32 (2.4G2, BD Bioscience, 1:200) and LIVE/DEAD™ Fixable Blue Dead Cell stain (Thermo Fisher Scientific, 1:500). Following washing, cells were stained at 4 °C for 25 min with rat anti-FCRLS (1:300 [66], provided by Oleg Butovsky) or rat anti-4D4 (1:300 [40]), followed by staining at 4 °C for 20 min with secondary goat anti-rat IgG-APC (Poly4054, Biolegend, 1:300). Cells were washed and then incubated with mouse anti-MHC-I (H-2 Kb/H-2D^b^)-PE (28-8-6, Biolegend, 1:100) and rat anti-CD11b-PerCP/Cy5.5 (M1/70, BD Bioscience, 1:200) at 4 °C for 30 min. Cells were then fixed in 4% PFA prior to analysis performed on an LSR-II flow cytometer (BD Bioscience) and data were analyzed with FlowJo software version 10 (BD Bioscience).

### Computational analysis of flow cytometry data

Computational analysis of flow cytometry data was performed using the Spectre R package [67] (package publicly available: https://github.com/ImmuneDynamics/Spectre). Live CD45^+^ and/or CD11b^+^ cells were manually gated and exported as CSV-channel files. Keywords denoting the sample and group names were then added to the samples, before the samples were merged into a single data table. The Flow Self-Organizing Maps (FlowSOM) algorithm [68] was then run on the merged dataset to cluster the dataset, where every cell is assigned to a specific cluster. The data were downsampled so that the relative number of cells in each sample was represented proportionally. Subsequently, the downsampled data were analyzed by the dimensionality reduction algorithm Uniform Manifold Approximation and Projection (UMAP) [69] for cellular visualization. Following FlowSOM clustering and dimensionality reduction with UMAP, summary tables containing expression level, cell frequency and cell number data of both the large FlowSOM and smaller UMAP datasets were exported and the total cell number of each cell type cluster was calculated.

### Statistics

Results are presented as individual values per mouse and mean ± SEM. The specific statistical tests used to determine significance are indicated in the figure legends. Statistical calculations were performed using Prism version 9 (GraphPad Software). For all data comparisons, a p-value < 0.05 was considered statistically significant.

## Results

### Activation of specific cytokine signaling pathways in the brain of GFAP-IL6 and GFAP-IFN mice

Both IL-6 and IFN-α signal via the JAK/STAT signal transduction pathway [70, 71], with canonical IL-6 signaling principally mediated through tyrosine 705 phosphorylated STAT3 (pY705-STAT3), whereas both tyrosine 701 and serine 727 phosphorylation of STAT1 (pY701-STAT1 and pS727-STAT1) are required for maximal activation of the IFN-α response [72, 73]. To assess stimulus-specific responses, we first performed immunoblots for phosphorylated STAT3 and STAT1 on cerebellum from GFAP-IL6 and GFAP-IFN mice (Additional file 1: Figs. S1, S2) since transgene expression of both cytokines is highest in this region compared with other areas of the brain [7, 8, 15, 74]. In agreement with previous reports [17, 75], GFAP-IL6 mice had high levels of pY705-STAT3 and low levels of pY701- and pS727-STAT1 in the cerebellum, while the cerebellum of GFAP-IFN mice had low levels of pY705-STAT3 and high levels of pY701- and pS727-STAT1 (Additional file 1: Fig. S1a). We next performed dual-label immunohistochemistry/histochemistry for pY705-STAT3 or pY701-STAT1 with tomato lectin, a microglia marker. In the cerebellum of WT mice, pY705-STAT3 and pY701-STAT1 were not detectable (Additional file 1: Fig. S1b, c). By contrast, since all resident cell types in the CNS are capable of responding to IL-6 (via trans-signaling) and IFN-α [2], the cerebellum of GFAP-IL6 and GFAP-IFN mice contained lectin-positive microglia and other, lectin-negative CNS-resident cells, with nuclear pY705-STAT3 or pY701-STAT1, respectively (Additional file 1: Fig. S1d–g). Importantly, GFAP-IL6 microglia had strong nuclear staining for pY705-STAT3, while nuclear pY701-STAT1 was not detected (Additional file 1: Fig. S1d, e). On the other hand, GFAP-IFN microglia had strong nuclear staining for pY701-STAT1, while nuclear pY705-STAT3 was not detected (Additional file 1: Fig. S1f, g). These findings highlight the remarkable signaling specificity of microglia in response to the neuroinflammation induced by chronic IL-6 versus IFN-α production, despite the pleiotropic effects of these cytokines and the secondary inflammatory factors which are induced by the chronic neuroinflammation in these animals. Together, our findings are consistent with microglia mounting stimulus-specific responses to transgene-driven production of IL-6 versus IFN-α. Further, while both GFAP-IL6 and GFAP-IFN mice exhibited robust microgliosis, with increased lectin binding compared with WT (Additional file 1: Fig. S1b-g), these cells also appeared to exhibit dramatic differences in number and morphology. Therefore, we next aimed to precisely dissect the molecular and cellular changes made by microglia in response to chronic IL-6 versus IFN-α production in the brain.

### Microglia of GFAP-IL6 and GFAP-IFN mice have distinct turnover patterns

**Fig. 1:**
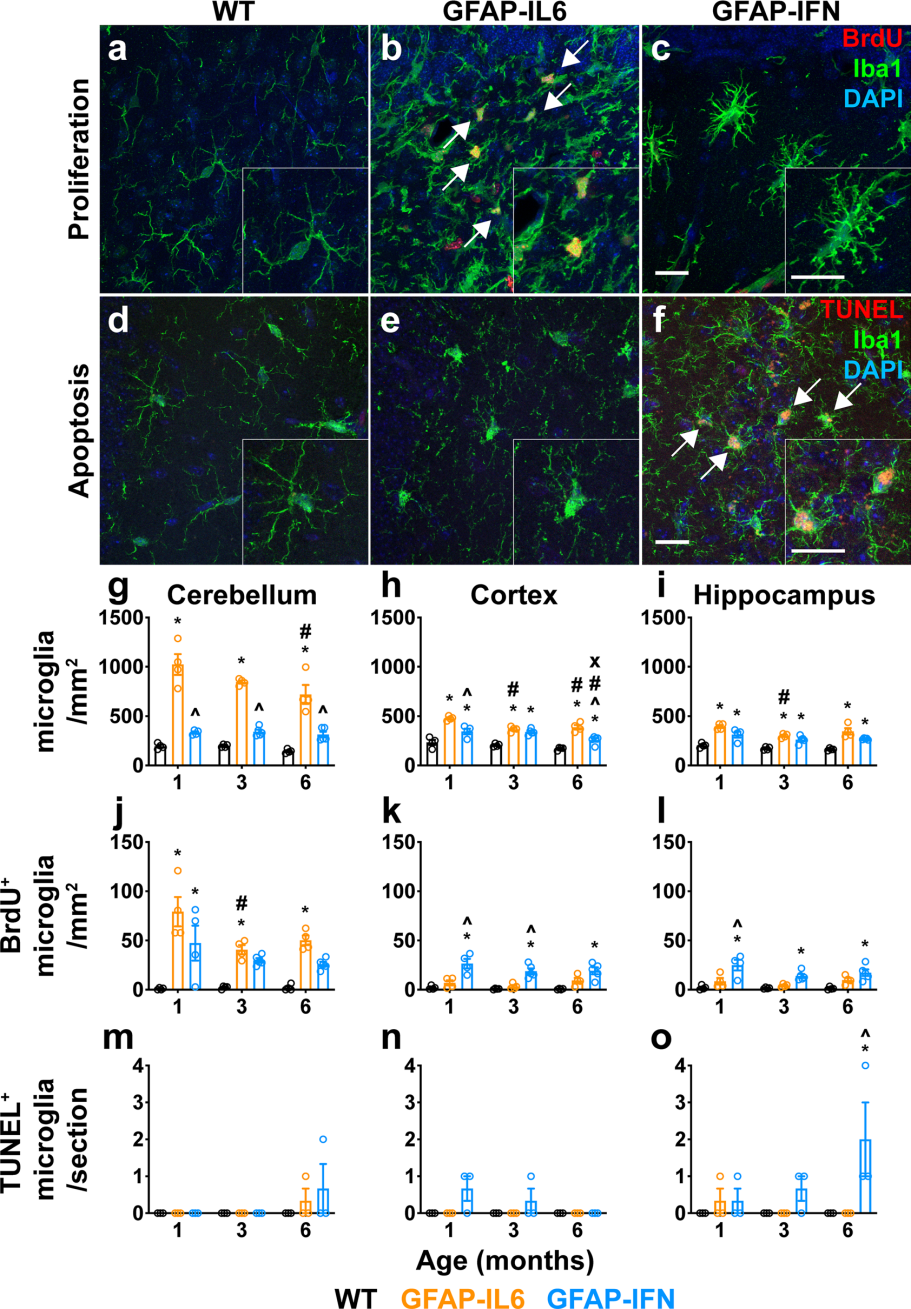
Microglia in the brain of GFAP-IL6 versus GFAP-IFN mice have unique turnover patterns. **a–c** Representative immunofluorescence images (Iba1^+^ microglia, green; BrdU^+^, red; DAPI, blue) from the cerebellum of 1-month-old WT (**a**), GFAP-IL6 (**b**) and GFAP-IFN (**c**) mice. **d–f** Representative images (Iba1^+^ microglia, green; TUNEL^+^, red; DAPI, blue) from the hippocampus of 6-month-old WT (**d**), GFAP-IL6 (**e**) and GFAP-IFN (**f**) mice. Scale bars, 20 μm. **g–o** Quantification of the total number of Iba1^+^ microglia per mm^2^ (**g-i**), the number of Iba1^+^BrdU^+^ microglia per mm^2^ (**j–l**) and the number of Iba1^+^TUNEL^+^ apoptotic microglia per section (**m–o**) in the cerebellum (**g, j, m**), cortex (**h, k, n**) and hippocampus (**i, l, o**) at 1, 3 and 6 months of age. *n* = 3–5 mice/group. Graphs show individual values per mouse and mean ± SEM. *, p < 0.05 compared with WT of the same age; ^, p < 0.05 compared with GFAP-IL6 of the same age; #, p < 0.05 compared with 1-month-old of the same genotype; x, p < 0.05 compared with 3-month-old of the same genotype using two-way ANOVA with Tukey’s post-test

Given the apparent stimulus-specific cytokine modulation of microglia cell numbers in the brains of GFAP-IL6 versus GFAP-IFN mice, we questioned whether there were changes to microglia turnover in response to chronic IL-6 versus IFN-α production. Indeed, the number of microglia in the cerebellum, cortex and hippocampus of the GFAP-IL6 mice was increased compared with both WT and GFAP-IFN mice at all ages studied (Fig. 1a–i). Although transgene expression of IL-6 is lower in the cortex and hippocampus than the cerebellum [7, 8, 15, 74], GFAP-IL6 mice still had significantly increased numbers of microglia in these regions compared with WT mice (Fig. 1h–i). Notably, GFAP-IL6 mice had significantly greater numbers of microglia compared with WT mice at all ages. However, the number of microglia decreased with age, with significantly fewer cells in the cortex and hippocampus of 3-month-old mice and significantly fewer cells in the cerebellum and cortex of 6-month-old mice compared with 1-month-old GFAP-IL6 mice. In contrast, microglia numbers in the cerebellum of GFAP-IFN mice were slightly, but not significantly, increased compared with WT mice and these numbers remained largely unchanged at all ages studied. The density of microglia in discrete brain regions also differed by genotype. Significantly greater numbers of microglia were seen in the cerebellum of GFAP-IL6 mice than in the cortex and hippocampus (Fig. 1g–i). By contrast, the density of microglia was roughly equal in the cerebellum, cortex and hippocampus of WT and GFAP-IFN mice, with the exception of the hippocampus of 3-month-old GFAP-IFN mice, which had significantly less microglia compared with the cerebellum and cortex.

To determine the basis for the differences in microglia numbers between mouse lines and age, we assessed proliferation and apoptosis rates. In all three brain regions of WT mice, the numbers of proliferative microglia as evaluated by BrdU incorporation and immunostaining (Fig. 1j–l) and apoptotic microglia as evaluated by TUNEL staining were very low (Fig. 1m–o). There were significantly increased numbers of BrdU^+^ microglia in the cerebellum of GFAP-IL6 mice as compared with WT mice at all ages (Fig. 1j). Interestingly, at later ages, the cerebellum of GFAP-IL6 mice contained significantly fewer BrdU^+^ microglia as compared with 1-month-old GFAP-IL6 mice. There were also low numbers of BrdU^+^ microglia in the cortex and hippocampus (Fig. 1k, l), consistent with lower numbers of total microglia in these regions in GFAP-IL6 mice (Fig. 1g–i). In addition, there was no significant increase in the number of TUNEL^+^ microglia in the brain of GFAP-IL6 mice (Fig. 1m–o). On the other hand, while the cerebellum of 1-month-old GFAP-IFN mice had comparable numbers of BrdU^+^ microglia to 1-month-old GFAP-IL6 mice, there was a small, but not statistically significant, increase in the number of BrdU^+^ microglia in the cerebellum of GFAP-IFN mice at later ages (Fig. 1j). Furthermore, in the cortex and hippocampus, GFAP-IFN mice had significantly increased numbers of BrdU^+^ microglia at all ages examined compared with WT mice (Fig. 1k, l). In addition, GFAP-IFN mice had a progressive increase in the number of TUNEL^+^ microglia, particularly in the hippocampus (Fig. 1m–o). Taken together, these findings indicate that the increase in microglia number and density in GFAP-IL6 mice is accounted for by increased proliferation but not apoptosis. On the other hand, despite increased proliferation, the number and density of microglia in GFAP-IFN mice are relatively unchanged, likely due in part to increased apoptosis.

### Microglia in the brains of GFAP-IL6 and GFAP-IFN mice exhibit unique morphological changes in response to chronic IL-6 versus IFN-α production

**Fig. 2:**
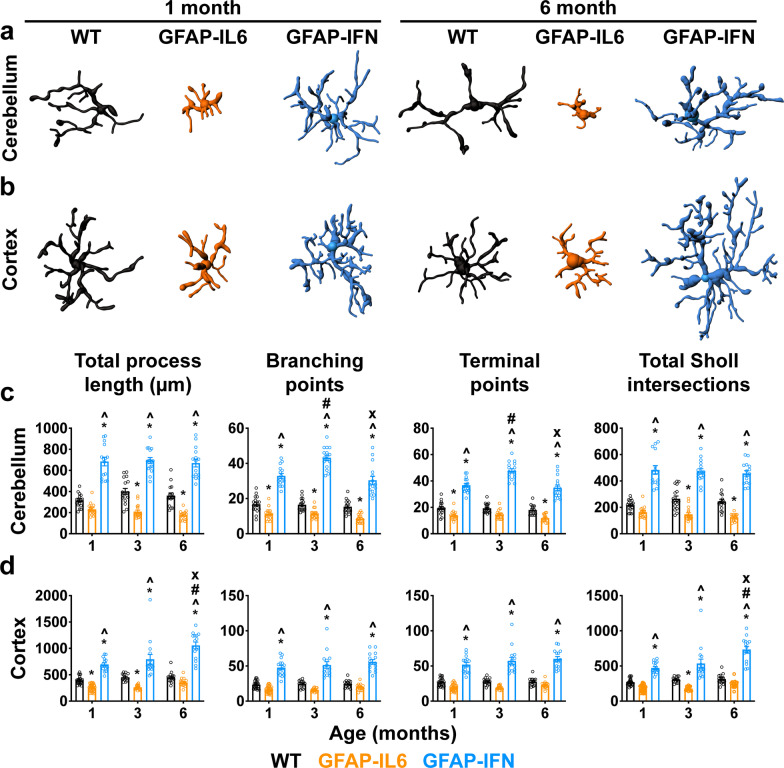
Microglia have distinct morphologies in GFAP-IL6 versus GFAP-IFN mice. **a-b** Representative three-dimensional reconstructions of Iba1^+^ microglia from the cerebellum (**a**) and cortex (**b**) of 1- and 6-month-old WT, GFAP-IL6 and GFAP-IFN mice. **c-d** Imaris automated quantification of the total process length, branching points, terminal points and total Sholl intersections of cerebellar (**c**) and cortical (**d**) microglia. *n* = 2–3 mice/group, *n* = 15–31 cells/genotype. Graphs show individual values per cell and mean ± SEM. *, p < 0.05 compared with WT of the same age; ^, p < 0.05 compared with GFAP-IL6 of the same age; #, p < 0.05 compared with 1-month-old of the same genotype; x, p < 0.05 compared with 3-month-old of the same genotype using two-way ANOVA with Tukey’s post-test

Microglia morphology in GFAP-IL6 and GFAP-IFN mice appeared distinct (Fig. 1a–f, Additional file 1: Fig. S1b–g). To quantify morphological differences of microglia in our mouse models, we generated three-dimensional reconstructions of Iba1-immunostained microglia from the cerebellum and cortex of GFAP-IL6 and GFAP-IFN mice (Fig. 2a, b). Compared with WT microglia, the total process length of cerebellar and cortical GFAP-IL6 microglia, as well as the number of branching points, terminal points and the total number of Sholl intersections, were reduced (Fig. 2c, d). This was most pronounced in the cerebellum (Fig. 2c). On the other hand, in GFAP-IFN mice, cerebellar and cortical microglial cell total process length and the number of branching points, terminal points and the total number of Sholl intersections were significantly increased compared with both WT and GFAP-IL6 microglial cells at all ages (Fig. 2c, d). Taken together, the cytokine environments induced by chronic IL-6 versus IFN-α production in the brain of GFAP-IL6 versus GFAP-IFN mice cause microglia to adopt distinct morphological states.

### Divergent transcriptional landscapes arise in microglia from GFAP-IL6 versus GFAP-IFN mice

**Fig. 3:**
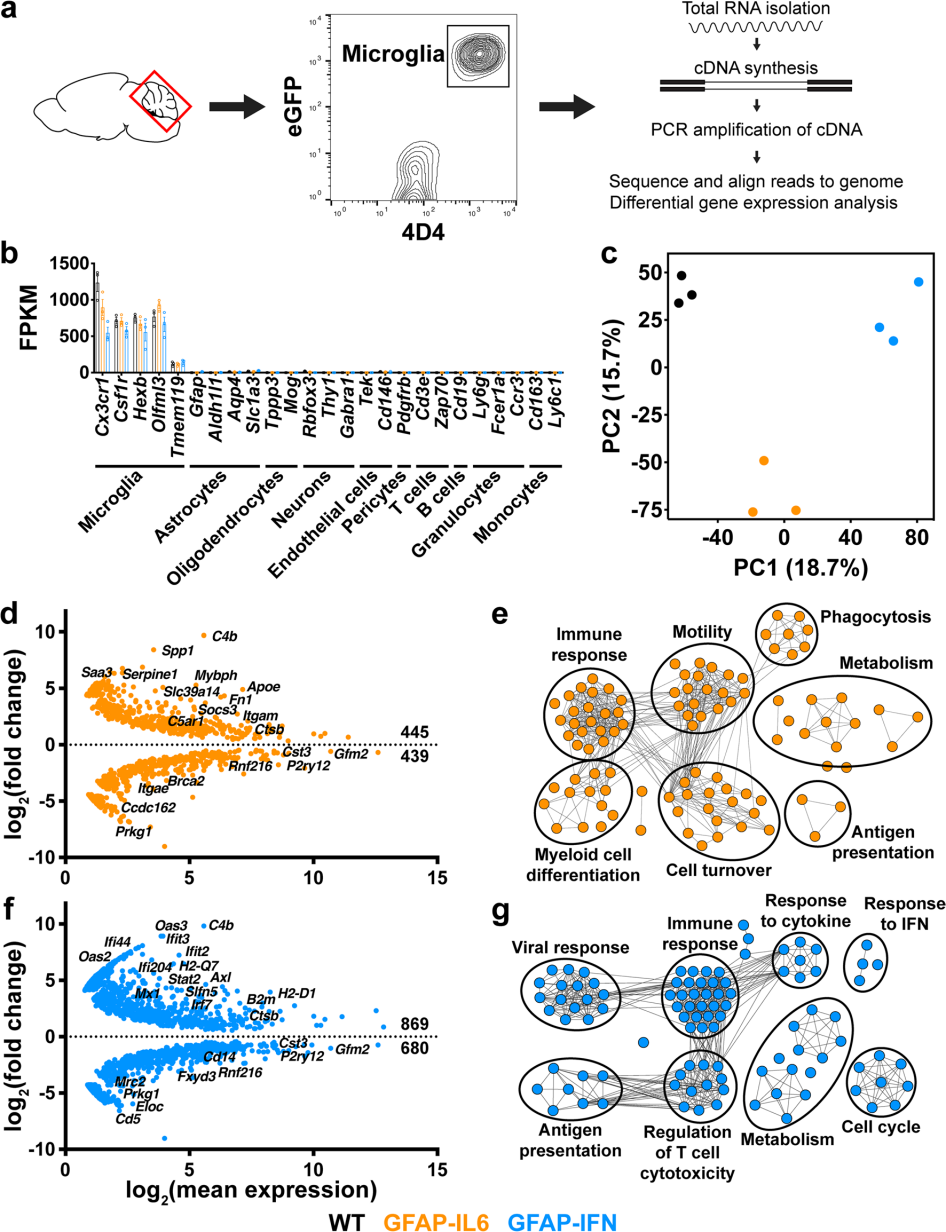
Cerebellar microglia regulate distinct subsets of genes in response to the cytokine environments induced by chronic IL-6 versus IFN-α signaling. **a** Microglia were isolated from the cerebellum of 1-month-old MacGreen-WT, -GFAP-IL6 and -GFAP-IFN mice and purified by FACS of live eGFP^+^ 4D4^+^ cells. RNA was isolated and reverse transcribed into cDNA, which was then amplified by PCR and sequenced. **b** Fragments per kilobase of transcript per million mapped reads (FPKM) of cell type-specific genes for microglia, other CNS-resident cells and peripheral leukocytes. **c** PCA of RNA-seq datasets of cerebellar microglia from WT, GFAP-IL6 and GFAP-IFN mice. **d** MA plot (representing log-ratio (M) on the y-axis and mean average (A) on the x-axis) showing transcripts differentially expressed by GFAP-IL6 cerebellar microglia compared with WT microglia. **e** Enrichment map of enriched GO biological processes by WebGestalt generated from the IL-6-regulated DEGs. **f** MA plot showing transcripts differentially expressed by GFAP-IFN cerebellar microglia compared with WT microglia. **g** Enrichment map of enriched GO biological processes by WebGestalt generated from the IFN-α-regulated DEGs. For **d, f**, the number of significantly (FDR < 0.05) upregulated and downregulated genes are indicated. For **e, g**, nodes in enrichment maps are significantly enriched in GO lists (FDR < 0.05) and were used to name clusters. *n* = 3 mice/group

To examine the effects of chronic IL-6 versus IFN-α signaling in the brain on the microglial cell phenotype on the transcriptional level, GFAP-IL6 and GFAP-IFN mice were crossed with MacGreen mice to label the myeloid compartment including microglia with eGFP [39]. We further used the microglia-specific 4D4 antibody, which does not bind to recruited monocytes/macrophages [40], to isolate and purify microglia from the cerebellum of 1-month-old mice by FACS of live dual-labeled eGFP^+^ 4D4^+^ cells and performed RNA-seq (Fig. 3a). We focused on this early age as the direct effects of IL-6 versus IFN-α are more discernible, since neuropathological changes including inflammation and neurodegeneration that arise because of chronic cytokine signaling are modest at this age. The purity of the microglial cell preparation was confirmed by robust expression of microglia signature genes, such as *Cx3cr1*, *Csf1r*, *Hexb*, *Olfml3* and *Tmem119*, while expression of genes specific for astrocytes, oligodendrocytes, neurons, endothelial cells, pericytes, T cells, B cells, granulocytes and monocytes was not detectable (Fig. 3b). We next performed principal component analysis (PCA) on all genes that passed the expression level criteria to examine the overall differences in the transcriptional landscape of cerebellar microglia (Fig. 3c). PCA showed that cerebellar microglia from WT, GFAP-IL6 and GFAP-IFN mice all had a high degree of separation from one another, indicative of highly divergent transcriptomes in these cells. Compared with WT microglia, microglia from GFAP-IL6 mice had 445 upregulated and 439 downregulated genes, with upregulation of genes classically associated with an IL-6-response, including *Spp1*, *Fn1*, *Socs3, Saa3* and *Apoe* (Fig. 3d, Additional file 2: Table S1). The IL-6-regulated differentially expressed genes (DEGs) were enriched in gene ontology (GO) biological processes such as immune response, myeloid cell differentiation, motility, cell turnover, phagocytosis, metabolism and antigen presentation (Fig. 3e). On the other hand, in response to the chronic production of IFN-α in the brain of GFAP-IFN mice, cerebellar microglia had 869 upregulated and 680 downregulated genes compared with WT cells, with upregulation of genes associated with responsiveness to IFN-α, including *Ifit3*, *Stat2*, *B2m*, *H2-Q7*, *Oas2* and *Slfn5* (Fig. 3f, Additional file 2: Table S1). The IFN-α-regulated DEGs were enriched in biological processes including immune response, viral response, response to IFN, response to cytokine, antigen presentation, regulation of T cell cytotoxicity, metabolism and cell cycle (Fig. 3g).

**Fig. 4:**
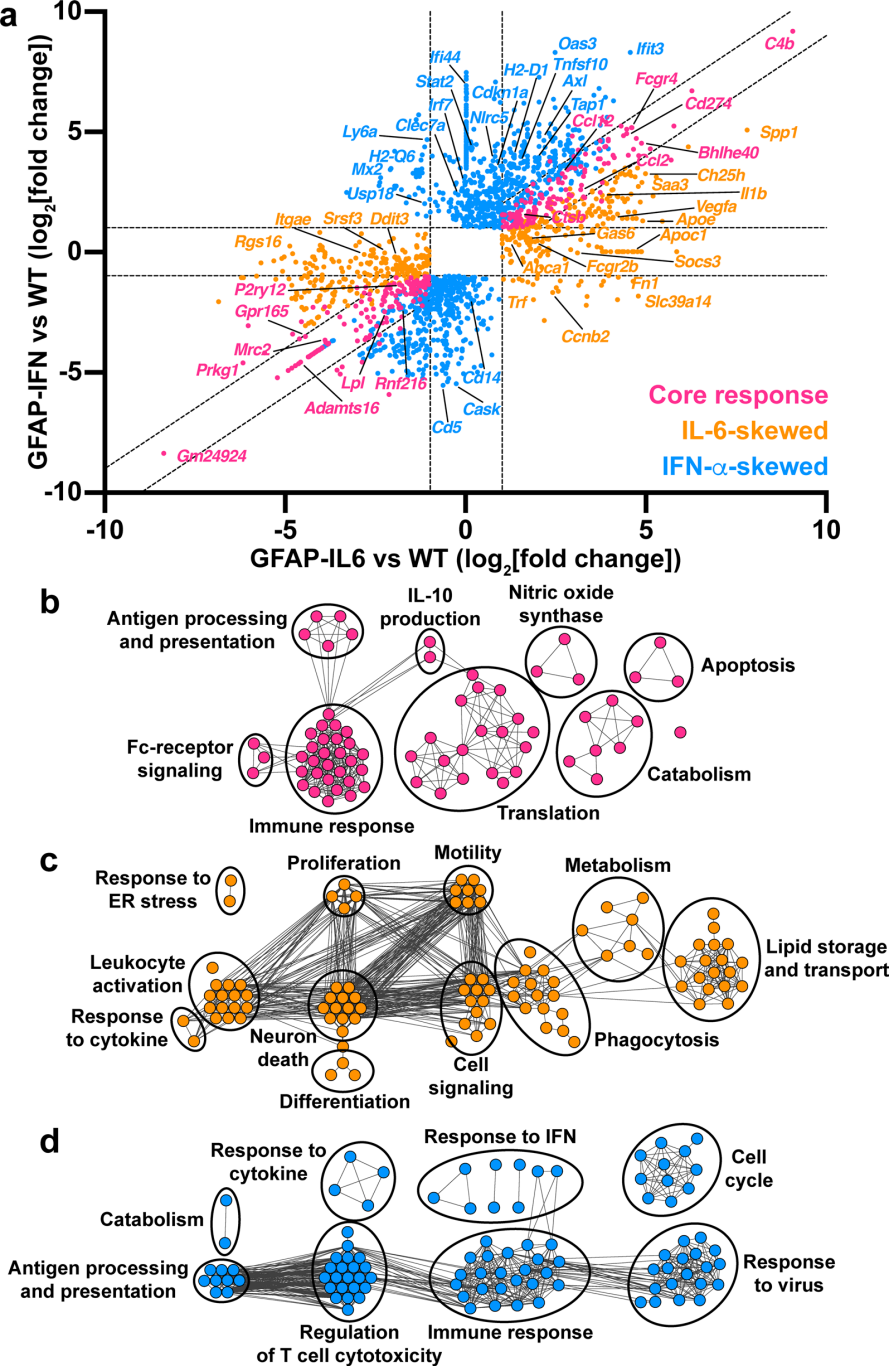
Microglia in GFAP-IL6 versus GFAP-IFN mice acquire unique transcriptional programs in addition to a common set of core response genes. **a** Two-way fold-change plot of differentially expressed genes in GFAP-IL6 versus WT microglia and GFAP-IFN versus WT microglia to identify core response genes (pink), as well as IL-6-skewed (orange) and IFN-α-skewed (blue) genes. **b** Enrichment map of top 100 significantly enriched GO biological processes by WebGestalt generated from the DEGs that are commonly upregulated by IL-6 and IFN-α. **c** Enrichment map of top 100 significantly enriched GO biological processes by WebGestalt generated from the DEGs that are upregulated and IL-6-skewed. **d** Enrichment map of top 100 enriched GO biological processes by WebGestalt generated from the DEGs that are upregulated and IFN-α-skewed. For **b–d**, nodes in enrichment maps are significantly enriched in GO lists (FDR < 0.05) and were used to name clusters

We next asked which DEGs are commonly regulated to a similar degree in response to IL-6 or IFN-α and which genes are IL-6- versus IFN-α-skewed. For this, we generated a two-way fold-change plot using genes at least twofold differentially expressed by GFAP-IL6 versus WT microglia and/or genes at least twofold differentially expressed by GFAP-IFN versus WT microglia (Fig. 4a) and performed GO analysis on the upregulated DEGs (Fig. 4b-d). There were no GOs that were significantly enriched by downregulated genes alone. Cerebellar microglia upregulated 144 genes and downregulated 143 genes to a similar degree in response to chronic production of IL-6 or IFN-α, with regulation of genes associated with microglia activation (upregulation of *C4b*, *Bhlhe40*, *Ccl2*, *Ccl12* and downregulation of *P2ry12*) (Fig. 4a, Additional file 3: Table S2). Interestingly, microglia from GFAP-IFN mice had a more pronounced and more extensive response than cells from GFAP-IL6 mice, since the overlapping core response genes comprised only 20.5% of the genes regulated by microglia in response to the IFN-α-induced cytokine environment, while core response genes comprised 37.0% of the genes regulated by microglia in response to the IL-6-induced cytokine environment (Additional file 1: Fig. S3). The core response genes upregulated by microglia from both transgenic mice were significantly enriched in processes including immune response, Fc-receptor signaling, antigen processing and presentation, IL-10 production, nitric oxide synthase biosynthesis, apoptosis, catabolism and translation (Fig. 4b). Since these biological processes are predominantly related to the modulation of the microglia immune response, these common transcriptional changes likely represent the overlapping target genes activated by both IL-6 and IFN-α, as well as the milieus induced by these cytokines within the brain tissue.

Two-way analysis also identified 252 upregulated and 237 downregulated genes that were exclusively regulated by IL-6 or were skewed such that they were significantly regulated by IL-6 compared with IFN-α and included *Spp1*, *Apoe*, *Saa3*, *Socs3*, *Fn1*, *Slc39a14* and *Ccnb2* (increased expression) and *Ddit3* and *Itgae* (decreased expression) (Fig. 4a, Additional file 3: Table S2). IL-6-skewed genes that were upregulated by GFAP-IL6 microglia were enriched for biological processes including leukocyte activation, response to cytokine, response to endoplasmic reticulum (ER) stress, motility, neuron death, differentiation, cell signaling and metabolism (Fig. 4c). Consistent with the presence of proliferating microglia in the brain of GFAP-IL6 mice (Fig. 1), genes regulated by GFAP-IL6 microglia were also enriched in processes associated with proliferation (*Ccnb2*, *Cdc42*, *Fn1*, *Spp1* and *Vegfa*) (Fig. 4a, c). In addition, genes that were associated with the microglial cell response to IL-6 were enriched in functional processes including phagocytosis and lipid processing (*Apoe*, *Apoc1*, *Abca1*, *Ch25h* and *Spp1*) and import of extracellular material such as iron (*Slc39a14* and *Trf*).

On the other hand, we identified 675 upregulated and 435 downregulated genes that were exclusively regulated by IFN-α or were skewed such that they were significantly regulated by IFN-α compared with IL-6 and included *Axl*, *Cdkn1a*, *Ifit3*, *Irf7*, *H2-D1*, *Stat2*, *Tap1* and *Usp18* (increased expression) and *Cd14* (decreased expression) (Fig. 4a, Additional file 3: Table S2). IFN-α-skewed genes that were upregulated by GFAP-IFN microglia were enriched for biological processes including response to cytokine, response to IFN, cell cycle, metabolism, response to virus, immune response, regulation of T cell cytotoxicity and antigen processing and presentation (Fig. 4d). Microglia from GFAP-IFN mice, but not GFAP-IL6 mice, had high levels of mRNA for MHC-I genes *H2-D1*, *H2-K1*, *H2-Q4*, *H2-Q6*, *H2-Q7*, *H2-T10*, *H2-T22*, *H2-T23*, as well as other genes associated with antigen processing, such as *B2m*, *Tap1* and *Tapbp* (Fig. 4a, d). Enhanced MHC-I expression may be induced by expression of the NOD-like receptor *Nlrc5*, the master regulator of MHC-I gene expression [76], which was also upregulated exclusively by GFAP-IFN microglia. Interestingly, consistent with the presence of proliferating and apoptotic microglia in the brain of GFAP-IFN mice (Fig. 1), biological processes associated with both cell cycle transition and apoptosis were significantly enriched by IFN-α-skewed genes. IFN-α-skewed genes included proliferation promoters (*Ccnd2*, *Ccnf*, *Cdc23*, *Ube2c* and *Kifc1*), as well as proliferation inhibitors (*Cdkn1a*, *Cdkn2c*, *Cdkn2d*, *E2f7* and *E2f8*) (Fig. 4a, d). Furthermore, GFAP-IFN microglia also expressed IFN-α-skewed genes that induce apoptosis (*Casp4*, *Tnfsf10*, *Pidd1*, *Shisa5*, *Ifit2*, *Ifit3*, *Oas1h* and *Rnasel*), as well as genes that promote survival (*Axl*, *Adar* and *Apip*).

Expression of selected core response, IL-6- and IFN-α-skewed genes, further analyzed by RTPCR of microglia from 1-, 3- and 6-month-old mice, for the most part, validated the RNA-seq data (Additional file 1: Fig. S4). Furthermore, the expression of some core response and IL-6- and IFN-α-skewed genes was further enhanced in older GFAP-IL6 and GFAP-IFN mice. In summary, microglia adopt a common transcriptional program and express overlapping target genes in response to the cytokine environments induced by both IL-6 and IFN-α, but also express stimulus-specific IL-6- or IFN-α-skewed genes.

### Microglia in the brain of GFAP-IL6 versus GFAP-IFN mice have distinct surface marker profiles related to the biological processes identified by transcriptomic analysis

**Fig. 5:**
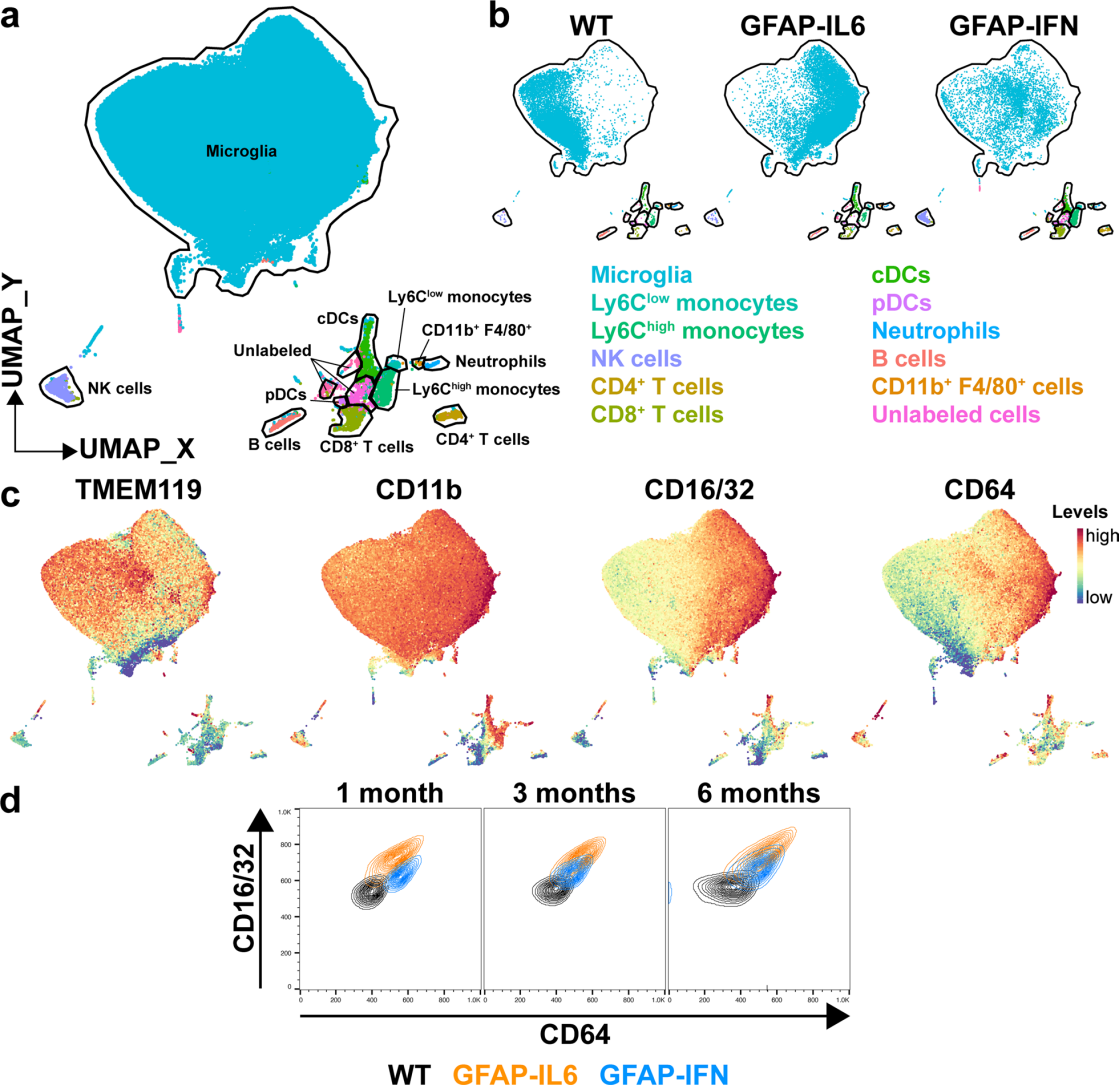
Distinct global leukocyte landscapes in the brain of GFAP-IL6 versus GFAP-IFN mice. **a** UMAP plot of entire dataset (brains of MacGreen-WT, -GFAP-IL6 and -GFAP-IFN mice at 1, 3 and 6 months of age) labeled with FlowSOM cluster identities. **b** UMAP plots of the 6-month-old dataset split into WT, GFAP-IL6 and GFAP-IFN mice. **c** UMAP plots of the same dataset colored by the expression of TMEM119, CD11b, CD16/32 or CD64. **d** Cluster overlay of CD16/32 versus CD64 levels on the surface of microglia from the brains of WT, GFAP-IL6 and GFAP-IFN mice at 1, 3 and 6 months of age. *n* = 3–5 mice/group

Having identified that microglia regulate unique genes associated with distinct functions in response to chronic IL-6 versus IFN-α production, such as phagocytosis and antigen presentation, we next examined whether these cells showed altered surface marker proteins associated with these cell functions. Since many of these functional markers are not specific to microglia, high-dimensional flow cytometry was performed on brain leukocytes from MacGreen-WT, MacGreen-GFAP-IL6 and MacGreen-GFAP-IFN mice and UMAP dimensionality reduction was first used to discern the global leukocyte landscape including microglia (Fig. 5, Additional file 1: Figs. S5, S6). UMAP analysis identified a large cluster of microglial cells as well as several leukocyte subpopulations present in the brain of the mice (Fig. 5a). Compared with WT, GFAP-IL6 mice had significantly increased numbers of conventional dendritic cells (cDCs) in the brain (Additional file 1: Fig. S6). On the other hand, compared with WT and GFAP-IL6 mice, there were increased numbers of Ly6C^low^ monocytes, Ly6C^high^ monocytes, NK cells, CD4^+^ T cells and CD8^+^ T cells present in the brain parenchyma of GFAP-IFN mice at all ages studied. In addition, the number of dendritic cells increased in the brain of GFAP-IFN mice with age and compared with WT and GFAP-IL6 mice, the number of cDCs in the brain of GFAP-IFN mice was significantly increased at 3 months of age and the number of plasmacytoid dendritic cells (pDCs) was significantly increased at 3 and 6 months of age.

Dimensionality reduction separated microglia by genotype, as WT, GFAP-IL6 and GFAP-IFN microglia occupied largely distinct areas of the microglia cluster, while genotype-dependent divergent clustering was not observed for other cell types (Fig. 5b). This suggests that microglia elicit robust, homogenous responses to chronic IL-6 and IFN-α production which drive these cells to adopt divergent states. Separation of microglial cells from WT, GFAP-IL6 and GFAP-IFN mice was based on differing surface levels of TMEM119, CD11b, CD16/32 and CD64 proteins (Fig. 5c). TMEM119 levels were lower on GFAP-IL6 microglia compared with WT and GFAP-IFN microglia. By contrast, levels of CD11b and CD16/32 were elevated on the surface of GFAP-IL6 microglia compared with WT and GFAP-IFN microglia. Additionally, CD64 levels were increased on microglia from both GFAP-IL6 and GFAP-IFN mice, with levels highest on the surface of GFAP-IL6 microglia. Two-way analysis of CD16/32 and CD64 levels demonstrated that microglia had differential responses in GFAP-IL6 versus GFAP-IFN mice (Fig. 5d). Compared with 1-month-old WT microglia, GFAP-IL6 microglia were CD16/32^hi^ CD64^hi^, while GFAP-IFN microglia had lower levels of CD16/32 than GFAP-IL6 microglia and were CD16/32^int^ CD64^hi^. Microglia in the brains of 3- and 6-month-old GFAP-IL6 mice remained CD16/32^hi^ CD64^hi^, however, cells in the brain of older GFAP-IFN mice had reduced levels of CD64 compared with 1-month-old GFAP-IFN mice.

**Fig. 6:**
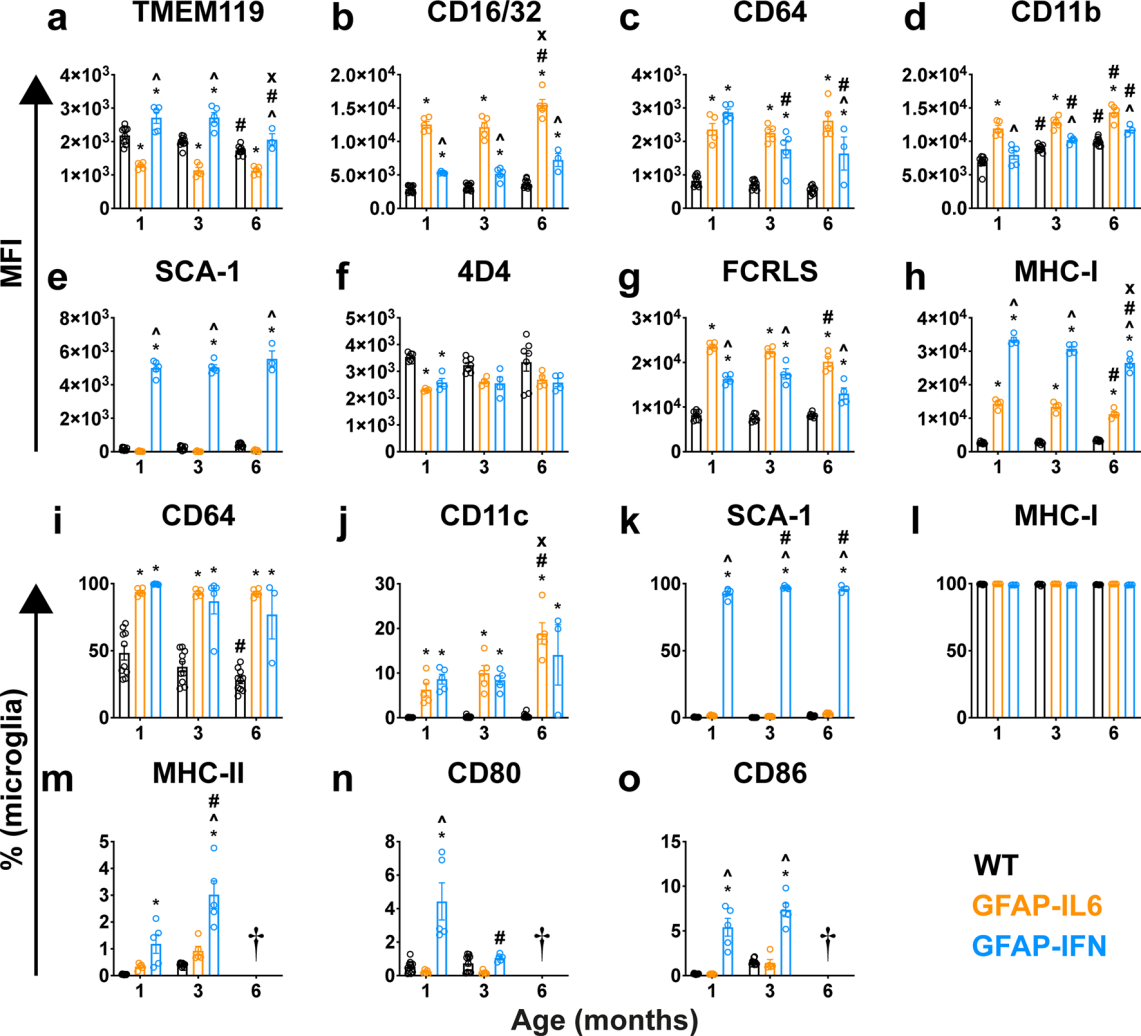
Microglia in the brain of GFAP-IL6 versus GFAP-IFN mice have unique surface marker expression profiles. **a–e** From 1-, 3- and 6-month-old MacGreen-WT, -GFAP-IL6 and -GFAP-IFN mice, microglia were gated (live eGFP^+^ CD45^low^ CD11b^+^ Ly6C^–^ TMEM119^+^ cells) and the median fluorescence intensity (MFI) was quantified for **a** TMEM119, **b** CD16/32, **c** CD64, **d** CD11b and **e** SCA-1 levels. **f–h** In a separate experiment, microglia were gated (live eGFP^+^ CD11b^+^ 4D4^+^ cells or live eGFP^+^ CD11b^+^ FCRLS^+^ cells) and the MFI was quantified for **f** 4D4, **g** FCRLS and **h** MHC-I levels. **i-o** Percentages of microglia positive for **i** CD64, **j** CD11c, **k** SCA-1, **l** MHC-I, **m** MHC-II, **n** CD80 and **o** CD86. *n* = 3–5 mice/group. Graphs show individual values per mouse and mean ± SEM. *, p < 0.05 compared with WT; ^, p < 0.05 compared with GFAP-IL6; #, p < 0.05 compared with 1-month-old of same genotype; x, p < 0.05 compared with 3-month-old of same genotype using two-way ANOVA with Tukey’s post-test. † N.B. The results for 6-month-old microglia are not shown due to high autofluorescence interference and are therefore not reliable

To further examine the microglial cell surface profile, we next used the data from Fig. 5 and gated for microglia (live eGFP^+^ CD45^low^ CD11b^+^ Ly6C^–^ TMEM119^+^ cells; Additional file 1: Fig. S7a) and quantified the median fluorescence intensity (MFI) of microglia signature and myeloid lineage markers (Fig. 6a–e), as well as the percentage of microglia that were positive for these markers (Fig. 6i-k, m–o). In a separate experiment, we gated microglia (live eGFP^+^ CD11b^+^ 4D4^+^ cells or live eGFP^+^ CD11b^+^ FCRLS^+^ cells; Additional file 1: Fig. S7b-c) and quantified the percentage of MHC-I^+^ cells and the MFI of 4D4, FCRLS and MHC-I (Fig. 6f-h, l). GFAP-IL6 microglia had reduced TMEM119 levels compared with both WT and GFAP-IFN mice, while GFAP-IFN microglia had elevated levels of TMEM119 compared with WT and GFAP-IL6 mice (Fig. 6a). Levels of microglia-specific 4D4 were slightly reduced on 1-month-old GFAP-IL6 and GFAP-IFN microglia compared with WT, however, at older ages, microglia from all three genotypes had comparable 4D4 levels (Fig. 6f). On the other hand, levels of the scavenger receptor FCRLS were significantly elevated on GFAP-IL6 and GFAP-IFN microglia compared with WT at all ages studied, however, these levels were highest on GFAP-IL6 microglia, being approximately 1.4-fold higher than that in GFAP-IFN mice (Fig. 6g).

Next, we examined the levels of several myeloid functional markers on the surface of microglia from GFAP-IL6 and GFAP-IFN mice. As demonstrated above, compared with WT, both GFAP-IL6 and GFAP-IFN microglia had significantly elevated levels of CD16/32 at all ages studied (Fig. 6b). However, surface CD16/32 levels on GFAP-IL6 microglia were 2.3-fold higher than those from GFAP-IFN mice and continued to increase with age. Due to low levels, only half of the microglia from the brain of WT mice were detected as CD64^+^ (Fig. 6i). By contrast, surface CD64 levels were significantly elevated to comparable levels on GFAP-IL6 and GFAP-IFN microglia at 1 month of age (Fig. 6c) and almost all microglia in the brain of these mice were CD64^+^ positive (Fig. 6i). While CD64 levels on GFAP-IL6 microglia remained elevated at all ages studied, levels on GFAP-IFN microglia gradually decreased with age, such that microglia from 6-month-old GFAP-IFN mice had CD64 levels 1.5-fold lower than GFAP-IL6 microglia (Fig. 6c). At all ages examined, microglia in the brain of GFAP-IL6 mice had significantly higher levels of CD11b compared with WT and GFAP-IFN mice (Fig. 6d). While CD11c^+^ microglia were barely detected in the brain of WT mice (Fig. 6j), the brain of both GFAP-IL6 and GFAP-IFN mice had a similarly sized CD11c^+^ microglia subpopulation that accounted for approximately 7% of microglia at 1 month of age, 10% of microglia at 3 months of age and 15% of microglia at 6 months of age.

GFAP-IFN microglia had increased surface levels of markers associated with IFN-α signaling, antigen presentation and lymphocyte co-stimulation, such as stem cell antigen-1 (SCA-1), MHC-I, MHC-II, CD80 and CD86 (Fig. 6e, h, k, l, m–o). Consistent with a robust microglial cell IFN-I response, virtually all microglia in the brain of GFAP-IFN mice were SCA-1^+^ (Fig. 6k). Although almost all microglia from all three genotypes were MHC-I^+^ (Fig. 6l), MHC-I levels were elevated by GFAP-IL6 and GFAP-IFN microglia compared with WT microglia (Fig. 6h). At all ages examined, MHC-I levels were highest on the surface of GFAP-IFN microglia and were 2.3-fold higher than levels of MHC-I on the surface of GFAP-IL6 microglia. The brain of WT mice had small numbers of MHC-II^+^ microglia (Fig. 6m). While 0.3% and 0.9% of 1- and 3-month-old GFAP-IL6 microglia, respectively, were MHC-II^+^, this increase was not significant. By contrast, the MHC-II^+^ microglial cell population was significantly increased in GFAP-IFN mice compared with WT, with 1.2% and 3% of microglia positive for MHC-II at 1 and 3 months of age, respectively. Additionally, the number of MHC-II^+^ microglia in the brain of 3-month-old GFAP-IFN mice was 2.5-fold higher than in 1-month-old GFAP-IFN mice. Only 0.5% and 0.2% of microglia were CD80^+^ in the brains of 1-month-old WT and GFAP-IL6 mice, respectively (Fig. 6n). By contrast, the proportion of microglia that were CD80^+^ in the brain of GFAP-IFN mice was 8.9-fold and 22-fold higher, respectively, at 1 month of age. Compared with 1-month-old GFAP-IFN mice, 3-month-old GFAP-IFN mice had a CD80^+^ microglia population that was approximately 4.2-fold smaller. Similarly, while a very small number (0.2–1%) of WT and GFAP-IL6 microglia were CD86^+^, at 1 and 3 months of age, there was a significant increase (30- and 5-fold, respectively) in the number of CD86^+^ microglia in the brain of GFAP-IFN mice (Fig. 6o). In summary, our findings demonstrate that the global leukocyte landscape is divergent in the brain of WT, GFAP-IL6 and GFAP-IFN mice and microglia adopt distinct surface marker profiles in these animals.

### IL-6- and IFN-α-like microglia responses are present in distinct neuropathological states

**Fig. 7:**
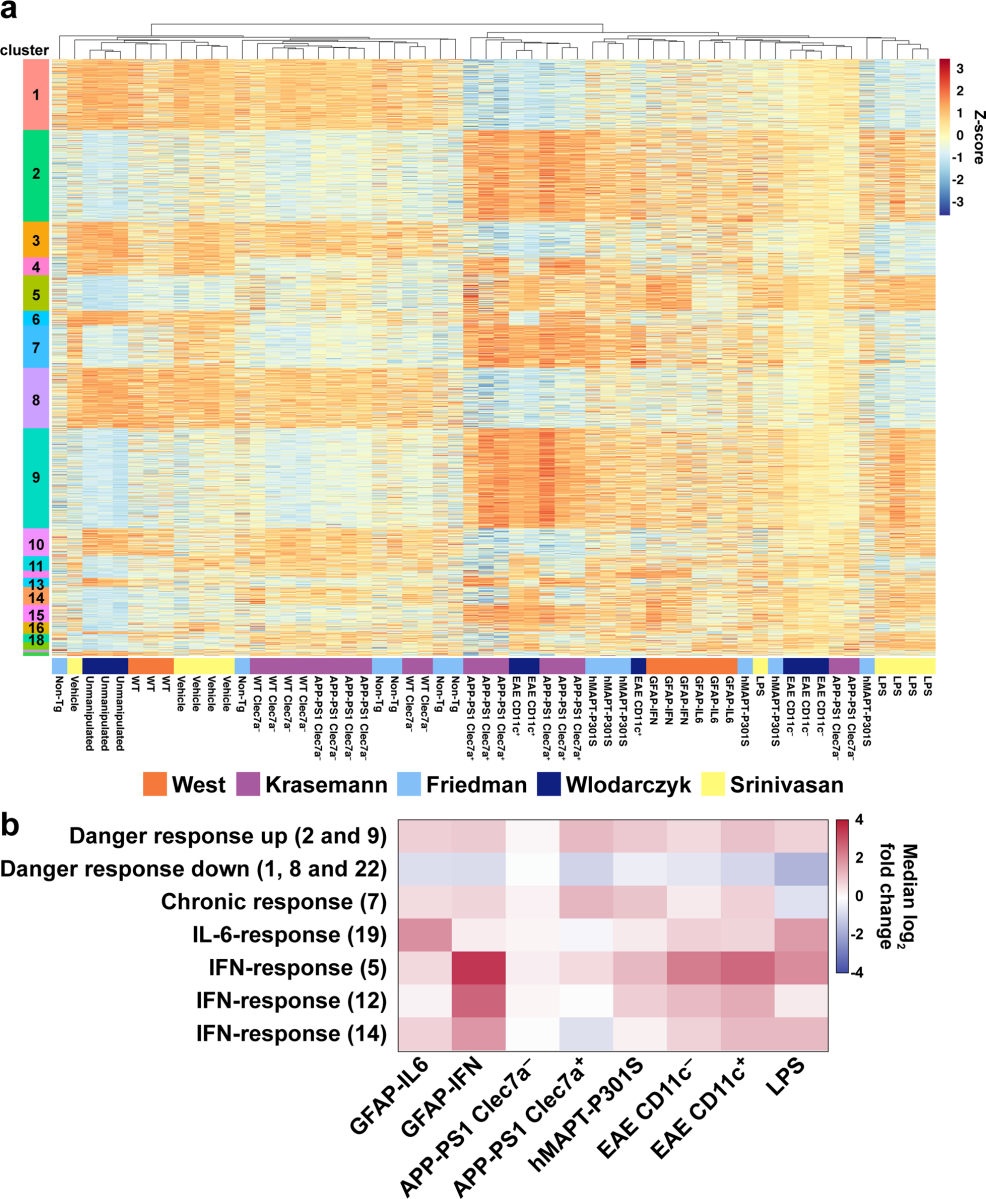
Meta-analysis of microglia gene expression datasets identified core and cytokine-specific co-regulated gene clusters. **a** Heatmap of z-scores (within-study-normalized) for 1,759 genes differentially expressed in at least 4 comparisons. Hierarchical clustering identified 22 clusters of co-regulated genes. **b** Summary of gene set changes from the meta-analysis, which identified upregulated danger response genes (clusters 2 and 9), downregulated danger response genes (clusters 1, 8 and 22), chronic response genes (cluster 7), IL-6-response genes (cluster 19) and IFN-response genes (clusters 5, 12 and 14). Differential expression was calculated by comparing each condition with its respective control. The median log_2_-fold change for each gene cluster is shown

We next asked whether microglia from other neuropathological states exhibited responses that aligned with those observed in the GFAP-IL6 and GFAP-IFN mice. We selected microglia RNA-seq studies reported for a number of different neurological diseases in mice, including Alzheimer’s disease (AD) [52], tauopathy [53], experimental autoimmune encephalomyelitis (EAE) [54] and lipopolysaccharide (LPS) endotoxemia [55]. In some disease models, microglia were isolated into subsets based on expression of disease-associated markers on their cell surface, such as Clec7a in AD [52] and CD11c in EAE [54]. Unfortunately, at the time of analysis, there were no appropriate RNA-seq datasets from purified microglia from mice during viral infection that we could include for comparison in our meta-analysis. Hierarchical clustering separated the samples in our gene expression matrix into a large cluster of microglia samples from “control” groups and a large cluster of microglia samples from “disease” groups (Fig. 7a, Additional file 1: Fig. S8). Additionally, there were 1,759 genes differentially expressed in at least 4 different conditions analyzed, which were separated into 22 co-regulated clusters by hierarchical clustering (Additional file 1: Fig. S8, Additional file 4: Table S3).

Using hierarchical clustering and visual inspection, we identified gene clusters 1, 2, 8, 9 and 22 that were dysregulated in almost every disease state and termed them "danger response genes". They included upregulated genes such as *Axl*, *Bhlhe40*, *C1qa* and *Spp1* and downregulated genes such as *Gpr34*, *Klk9*, *P2ry12* and *Rnf216* (Fig. 7b, Additional file 1: Fig. S9a–d, Additional file 4: Table S3). While APP-PS1 Clec7a^–^ microglia, which are not associated with Aβ-plaques [52], did not differentially regulate these genes, clusters 2 and 9 were broadly upregulated and clusters 1, 8 and 22 were broadly downregulated by microglia in the other neurological disease states analyzed, including APP-PS1 Clec7a^+^, hMAPT-301S, EAE CD11c^–^, EAE CD11c^+^ and LPS microglia. Interestingly, 50% of the genes identified by Friedman et al*.* in a microglia-specific cluster were identified as downregulated universal danger genes in our meta-analysis (Additional file 1: Fig. S10a, b) [53]. We also identified a cluster (cluster 7), which included 125 genes that were upregulated by microglia in response to chronic stimuli, such as neurodegeneration and autoimmunity, but were unchanged or decreased in microglia responding to acute LPS-induced endotoxemia; these genes were termed "chronic response genes" and included genes such as *Apoe*, *Clec7a*, *Gas6*, *Itgax* and *Siglecf* (Fig. 7b, Additional file 1: Fig. S9e-f, Additional 1: Table S3). While microglia not associated with pathology in APP-PS1 mice (APP-PS1 Clec7a^–^ microglia) did not upregulate the chronic response genes, these genes were upregulated by APP-PS1 Clec7a^+^, hMAPT-P301S, EAE CD11c^–^ and EAE CD11c^+^ microglia. While many of the danger response and chronic response genes were also dysregulated by GFAP-IL6 and GFAP-IFN microglia, fold-change analysis demonstrated that the distribution of gene expression was more variable than in microglia from other neuropathological conditions (Additional file 1: Fig. S9b, d, f). This is likely the result of the modest neuropathological features of disease in these mice at 1 month of age, compared with the more pronounced perturbation of microglia in mice at later stages of disease in the other neuropathological states. However, many of the danger response and chronic response genes upregulated by microglia in various different neuropathological states were classified by us as either core response genes commonly regulated by IL-6 and IFN-α, or IL-6- or IFN-α-skewed, with genes that are regulated by microglia in response to IL-6 and/or IFN-α comprising 36.1%, 35.8% and 30.4% of the danger response gene clusters 2 and 9, clusters 1, 8 and 22 and the chronic response gene cluster 7, respectively (Additional file 1: Fig. S10c–e).

In addition to core transcriptional programs, meta-analysis also identified cytokine-specific gene signatures that were only regulated in certain disease states (Fig. 7, Additional file 1: Fig. S11, Additional file 4: Table S3). Cluster 19, termed the "IL-6-response gene" cluster, consisted of 22 genes, including *Ccnb2*, *C5ar1*, *Hs3st3b1 and Id2*, which were highly upregulated in microglia from GFAP-IL6 mice and mice with LPS-induced endotoxemia (Fig. 7b, Additional file 1: Fig. S11a, e), with the median log_2_ expression of these genes upregulated 2.1- and 1.9-fold, respectively, compared with their respective controls. Similarly, these genes were modestly upregulated in both CD11c^–^ and CD11c^+^ microglia during EAE, with the median log_2_ expression of these genes upregulated 0.9- and 0.8-fold respectively. By contrast, in neurodegenerative conditions such as AD and tauopathy, microglial cell expression of the IL-6-response cluster genes were unchanged or only slightly increased.

Hierarchical clustering also identified three clusters, clusters 5, 12 and 14, which included a total of 179 genes; these genes were termed "IFN-response genes" and included *Adar*, *H2-Q7*, *Ifit1*, *Irf7*, *Tnfsf10* and *Gbp2* (Fig. 7b, Additional file 1: Fig. S11b-d, f–h, Additional file 4: Table S3). In addition to GFAP-IFN microglia, most of the IFN-response cluster genes were highly upregulated in both CD11c^–^ and CD11c^+^ microglia during EAE and in microglia during LPS-induced endotoxemia. On the other hand, regulation of the IFN-response genes by microglia from GFAP-IL6 mice and in neurodegenerative states, such as Clec7a^+^ cells from APP-PS1 mice and microglia from hMAPT-301S mice, was more variable, with only modest upregulation of IFN-response genes in some clusters and unchanged or downregulated expression in others. Taken together, in distinct neuropathological states, microglia uniquely regulate genes associated with IL-6 or IFN-α signaling, which are components of the universal danger response genes and, in chronic states of perturbation, the chronic microglia response.

## Discussion

Here we examined the phenotypic changes of microglia in response to chronic IL-6 versus IFN-α signaling in the brain. In response to the respective cytokine milieu, microglia undergo distinct molecular and cellular adaptations as they fine-tune their phenotypes in the brain of GFAP-IL6 versus GFAP-IFN mice. Their adaptations correlated with the expression of unique IL-6- or IFN-α-skewed genes in addition to a core transcriptional program. Further, transcriptomic meta-analysis demonstrated that both IL-6 and IFN-α contribute to the formation of a core microglial cell transcriptional response in a wide range of neuropathological conditions. Finally, our findings suggest that IFN-α signaling has a more extensive effect on the microglial cell transcriptional landscape in disease compared with IL-6.

### Chronic IL-6 and IFN-α production induces distinct manifestations of neuroinflammation in GFAP-IL6 and GFAP-IFN mice

The distinct activation of the signature transcription factors STAT3 and STAT1 in the brains of GFAP-IL6 and GFAP-IFN mice further highlighted the specific nature of the response by microglia to IL-6 and IFN-α, respectively, and is consistent with previous reports [17, 75]. Interestingly, serine phosphorylation of STAT1, which is required for the maximal activation of this transcription factor [72, 73], was barely detectable in GFAP-IL6 brains further indicating the minor role that STAT1 activation has in the CNS of these mice [75]. By contrast, low levels of STAT3 activation by IFN-α were also previously observed in the CNS of GFAP-IFN mice [75] and may play a role in the modulation of the IFN-I response [77]. The direct comparison of the activation of IL-6 and IFN-α signaling pathways in the CNS of GFAP-IL6 and GFAP-IFN mice demonstrates the remarkable specificity by which these pathways are activated. Although these cytokines are pleiotropic, the activation of STAT3 versus STAT1 is unique in the CNS of GFAP-IL6 and GFAP-IFN mice. Furthermore, the chronic production of IL-6 and IFN-α causes progressive, destructive diseases that are associated with the production of secondary mediators of inflammation in these mice [7, 8, 12, 15, 16, 28, 29], however, the specific activation of STAT3 versus STAT1 suggests that IL-6 and IFN-α induce distinct manifestations of neuroinflammation.

### The dynamics of microglial cell proliferation and apoptosis are divergently altered by IL-6- versus IFN-α-induced neuroinflammation

Significantly increased numbers of microglia have been reported in the brain of adult GFAP-IL6 mice [14, 78–81]. Our findings here in juvenile and adult mice revealed that this increase was the consequence of significantly augmented proliferation of these cells in the brain while apoptosis was unchanged. This is consistent with reports that IL-6 can stimulate the proliferation of microglia in vitro [82] and in vivo [83, 84]. However, the total number of microglia in GFAP-IL6 mice, as well as the number of BrdU^+^ microglia, decreased from as early as 1 month of age correlating with IL-6 mRNA levels peaking in 1- and 3-month-old GFAP-IL6 mice before declining by 8 and 12 months of age [14], possibly due to astrocyte degeneration and loss [13]. The notion that chronic IL-6 signaling directly promotes microglia proliferation is also supported by our observation of greater numbers of proliferating microglia present in brain regions of GFAP-IL6 mice shown to have high levels of IL-6, such as the cerebellum, compared with lower numbers of proliferating cells in regions with lower levels of IL-6, such as the cortex and hippocampus [8, 15, 74]. Since apoptosis did not appear to substantially contribute to the turnover of microglia in older GFAP-IL6 mice, the mechanism by which microglial cell numbers decreased in the cerebellum of aged animals remains unknown but may be the result of microglial cell egress to other regions of the brain or due to activation of alternative cell death pathways.

In contrast to IL-6, IFN-α exhibits anti-proliferative effects in bone marrow-derived macrophages [85, 86] and elevated maternal levels of the type I IFN, IFN-β, contribute to arrested microglial cell proliferation in the CNS of newborn mice [87]. It was therefore surprising to observe increased numbers of BrdU^+^ microglia in all three brain regions of GFAP-IFN mice independent of age. In vesicular stomatitis virus-mediated encephalitis, astroglial and neuronal, but not microglial IFNAR signaling is required for the proliferation of microglia, suggesting that astrocytes and neurons respond to IFN-I and in turn regulate the ability of microglia to proliferate during viral encephalitis [88]. Thus, it is conceivable that in GFAP-IFN mice, astrocytes and neurons respond to high levels of IFN-α by producing factors which promote the proliferation of microglia. Importantly, TUNEL staining indicated that the increase in microglial cell number due to proliferation was offset by the loss of these cells due, at least in part, to increased apoptosis. Induction of apoptosis is a well-known action of IFN-I [89–91] and microglia from GFAP-IFN mice had significantly increased expression of a number of genes linked to the apoptosis-promoting function of IFN-α, including 2′-5′-oligoadenylate synthase (OAS), RNase L [92, 93], TRAIL (encoded by *Tnfsf10*) [94], interferon-induced protein with tetratricopeptide repeats (IFIT)2, IFIT3 [95] and the non-canonical inflammasome activator caspase-4 (encoded by *Casp4*) [96]. Thus, the cytokine environment induced by IFN-α in GFAP-IFN mice promotes both microglia proliferation and death, overall resulting in relatively stable cell numbers.

While the impact of microgliosis has been the subject of considerable attention, the functional outcomes of changes in microglial cell turnover in neurological diseases have largely been overlooked [97]. In mice intraperitoneally administered with LPS, there is transient, robust microglial cell proliferation in brain regions with substantial neuroinflammation [97]. Blockade of microglia proliferation in these animals prolonged the signs of LPS-induced sickness, demonstrating that the transient increase in microglia number is protective and these cells attenuate the sickness response [98]. Similarly, proliferating microglial cells are neuroprotective in cerebral ischemia and their selective ablation exacerbates neuroinflammation and ischemic damage [99]. On the other hand, the expansion of the microglial cell population during prion disease is detrimental and contributes to disease progression [100]. Together, these findings demonstrate that the regulation of the dynamics of microglia turnover is a critical component of the microglial cell response to neuroinflammation and can be helpful or harmful depending on the context.

### Neuroinflammation in GFAP-IL6 versus GFAP-IFN mice induces unique morphological changes in microglia

In addition to cell numbers, in the brain of GFAP-IL6 versus GFAP-IFN mice, microglia exhibited stimulus-specific morphological changes. Microglia from GFAP-IL6 mice had shorter processes with less branching and complexity, similar to earlier reports in these animals of microglia with larger somata and processes that occupy a smaller area of the brain [101]. Importantly, the microglia in GFAP-IL6 mice had a similar morphology to microglia in the brains of patients with NMOSD [24] and animals with pathologies associated with elevated IL-6, such as acute LPS-induced endotoxemia [102, 103], stroke [104] and ischemic stroke and reperfusion [105]. On the other hand, microglia from GFAP-IFN mice were hypertrophied, had longer processes and were hyper-ramified, comparable to microglia in the brain of mice reported during aging [106, 107], chronic stress [108, 109], IFN-α-induced depression [110] and following chronic, CNS-targeted production of IFN-β [107]. Furthermore, in neurodegenerative diseases such as AD, concurrent activation of IFN-I signaling [111–113] and the production of cellular injury- and death-associated factors and other AD-related proinflammatory factors, including IL-1β, IL-6 and TNF [112, 114], may contribute to the heterogenous appearance of classically “reactive” [112, 115] or hypertrophied microglia [111, 115, 116]. These findings further demonstrate that activated microglia do not follow a linear program towards an amoeboid morphology, but rather, microglia exist in a morphological spectrum that depends on the nature, duration and context of the stimuli [34]. Since the morphologies of microglia in GFAP-IL6 versus GFAP-IFN mice were similar to those observed in human diseases associated with elevated IL-6 versus IFN-α levels, it is conceivable that the microglial cell morphological changes observed in these diseases in humans reflect direct microglia responses to these cytokines.

### The distinct molecular signatures induced by chronic production of IL-6 versus IFN-α are associated with unique microglia activities

Paralleling the stimulus-specific differences in microglia turnover and morphology, we also observed stimulus-specific expression of genes and production of proteins in microglia from GFAP-IL6 versus GFAP-IFN mice. Correlating with progressive demyelination, proliferative angiopathy and iron accumulation observed in the brain of GFAP-IL6 mice [8, 13, 117], microglia expressed genes and proteins related to phagocytosis and processing of damaged myelin and other cellular detritus, as well as angiogenesis and the regulation and metabolism of iron. One of the most highly upregulated genes in GFAP-IL6 microglia, *Apoe*, is a core component of a neurodegenerative microglia phenotype (MGnD), also known as disease-associated microglia (DAM) transcriptional signature [52, 118]. Microglial cell secretion of APOE protein has been suggested to be a key player in the clearance of dead cells, extracellular debris and apoptotic neurons [119, 120], thereby enabling microglia to suppress damage to healthy neurons in neurodegenerative states [119]. However, APOE has been demonstrated to be an intrinsic regulator of a microglia phenotype associated with neurodegeneration and may contribute to disease [52, 121]. GFAP-IL6 microglia also uniquely upregulated *Vegfa*, which promotes angiogenesis and may contribute to the proliferative angiopathy that occurs in the brain of these animals [8]. VEGF-A also drives chemotaxis and proliferation of microglia and other cells [122], while LPS-activated microglia co-cultured with endothelial cells produce VEGF-A and promote angiogenesis [123]. Microglial cell expression of *Vegfa* in response to IL-6 may therefore induce both microglial cell proliferation and contribute to pathological angiogenesis observed in the brain of GFAP-IL6 mice [8]. Aberrant deposition of iron is a prominent feature of neurodegenerative diseases [124, 125] and progressively accumulates in the cerebellum of GFAP-IL6 mice, likely as a result of chronic leakage of the blood–brain barrier [117]. Increased microglial cell expression of IL-6-skewed genes associated with iron ion uptake and transport, such as *Slc39a14* and transferrin (*Trf*) [126], may therefore impart these cells with the ability to sense and take up iron in the brain of GFAP-IL6 mice. However, since iron accumulates in the brain of older GFAP-IL6 mice, this function is likely impaired, is not sufficient to restrain iron accumulation as these animals age, or iron remains in the brain following uptake by microglia.

In contrast to GFAP-IL6 mice, GFAP-IFN mice have unique brain pathology. The microglia in these animals predominantly regulated genes and surface marker proteins that were associated with antiviral immunity, with enrichment for processes related to antigen processing and presentation and regulation of T cell cytotoxicity. Microglia from GFAP-IFN mice had significantly increased levels of MHC-I and MHC-II, as well as the T cell co-stimulation molecules CD80 and CD86. The upregulation of antigen presenting-related markers, together with the hyper-ramified morphology of GFAP-IFN microglia, are indicative of the acquisition of an antigen-presenting cell phenotype, likely allowing these cells to communicate with, interact with and present antigen to peripheral leukocytes that progressively infiltrate the brain of these mice. These findings overlap with the transcriptomic profile of primary microglia stimulated with IFN-α in vitro [127] and are also consistent with heightened antiviral activity in the brain of GFAP-IFN mice, as indicated by improved survival and reduced immune pathology in lymphocytic choriomeningitis virus and herpes simplex virus-1-infected animals [7, 128].

### Microglia responses in neuroinflammatory and neurodegenerative diseases comprise IL-6- and IFN-α-like responses

Meta-analysis of the transcriptional landscapes of microglia in diverse neuropathological diseases including AD, tauopathy, EAE, LPS endotoxemia, as well as GFAP-IL6 and GFAP-IFN mice, identified a core microglial cell transcriptional response, termed danger response genes, which were similarly regulated in most of the disease states studied. These genes included *Axl*, *Bhlhe40*, *Cd274*, *Ctsb*, *Ctss*, *Spp1* and others, which were previously identified as widely regulated by microglia in many different conditions [53, 57, 129]. The upregulation of these genes allow microglia to react to perturbation, enabling them to phagocytose damaged cells and other material (*Axl*, *C1qa*, *C1qb*) [130–134], interact and communicate with recruited lymphocytes (*B2m*, *Cd274*, *H2-D1*) [135, 136], process and metabolize myelin debris and other detritus (*Ch25h*, *Lpl*) [137, 138], produce secondary inflammatory mediators (*Ccl3*, *Ctsb*, *Ctsc*, *Ctss, Trim25*) [139–144] and other functions. Furthermore, *Bhlhe40* was identified as a danger response gene and is suggested to be a putative transcription factor which regulates microglial cell activity in response to disturbances in their local environment [53]. In addition, danger genes universally downregulated by perturbed microglia included genes which distinguish microglia from myeloid cells in peripheral tissues. This is consistent with increasing evidence demonstrating that during neurological disease, microglia decrease expression of homeostatic signature genes and increase expression of neuroinflammatory genes in order to react to perturbation [34]. Chronic response genes that were upregulated in states of chronic neuroinflammation, but not in response to acute stimuli, included *Apoe*, *Cd83*, *Chst*2*, Clec7a*, *Itgax* and *Itgb2* and were similar to clusters of “primed” or “neurodegenerative” genes in other meta-analysis studies [53, 57]. Further, the chronic response of microglia to nucleic acid-positive amyloid, but not acute exposure to IFN-β, induced the expression of *Apoe* and *Clec7a* [112]. Importantly, a considerable number of the danger and chronic response genes were classified by us as IL-6- and/or IFN-α-regulated and comprised 36.1%, 35.8% and 30.4% of the danger response gene clusters 2 and 9, clusters 1, 8 and 22 and the chronic response gene cluster 7, respectively. These findings indicate that both IL-6 and IFN-α signaling, likely in combination with other inflammatory factors, have an important role in the formation of the core microglial cell transcriptional response that arises as a result of a wide range of pathogenic stimuli and/or loss of homeostasis.

We also identified clusters of cytokine-specific genes that were regulated in specific disease states. In both CD11c^–^ and CD11c^+^ microglia during EAE and in microglia during acute LPS-induced endotoxemia, IL-6- and IFN-response genes were highly enriched. However, these genes were upregulated to a lesser degree by microglia in neurodegenerative mouse models, such as APP-PS1 and hMAPT-P301S mice. Microglia have been shown to upregulate IFN-response genes in a number of distinct neuropathological conditions, including AD and LPS endotoxemia [53, 112, 145]. However, our meta-analysis identified multiple IFN-response gene clusters. Cluster 5 genes included the classical IFN-regulated genes *Adar*, *Ifit1*, *Irf7* and *Stat1* and these were upregulated to varying degrees by microglia in every disease state studied. On the other hand, microglia from GFAP-IFN mice and mice with EAE expressed high levels of IFN-response cluster 12 and 14 genes, while microglia in LPS endotoxemia only expressed high levels of cluster 14 genes and expression of cluster 12 and 14 genes was not changed or was instead downregulated in neurodegenerative conditions. Although, to our knowledge, comparative analysis of the levels of IL-6 or IFN-α in the CNS in multiple sclerosis (MS)/EAE versus AD has not been performed in humans or mice, our findings are indicative of a stronger, more extensive response made by microglia to IL-6 and IFN-I, likely due to higher levels of these cytokines in the CNS, during autoimmunity and acute insult compared with neurodegenerative diseases. Furthermore, the identification of one small cluster of IL-6-regulated genes and three large clusters of IFN-regulated genes, in addition to the larger overlap of IFN-skewed genes with danger and chronic response genes compared with IL-6-skewed genes, suggested that microglia elicit a more pronounced, extensive response to IFN-α compared with IL-6. In line with this, GFAP-IFN microglia had a larger total number of regulated genes and a smaller overlap of commonly regulated genes compared with GFAP-IL6 cells. These findings suggest that IFN-α may induce a stronger, dominant transcriptional response in disease states where both IL-6 and IFN-α are present, which may conceal or dilute the transcriptional effects of IL-6. This phenomenon should be considered when interpreting transcriptional profiles from neuroinflammatory diseases in which both IL-6 and IFN-α are present at elevated levels. For example, transcriptional analyses ascribe IFN-α with an important role in NMOSD [146, 147] but may under-appreciate the critical role of IL-6 signaling in these diseases. A prominence of both of these cytokines in NMOSD is consistent with clinical evidence, where IFN-β therapy exacerbates disease severity and increases relapse rates [148–152], in contrast to patients with MS where IFN-β ameliorates disease [153]. On the other hand, treatment with the IL-6R antibody tocilizumab significantly ameliorates disease in patients with NMOSD [148, 154–157].

### Limitations and future directions

Redundancy in the downstream effects of different cytokines must be considered when interpreting the results of this study. For example, chronic production of IL-6, IFN-α or TNF in the CNS of mice all promote a neuroinflammatory state which predisposes these animals to seizures and neurodegeneration [2, 158, 159]. Furthermore, transgenic production of IL-6 or IFN-α in the CNS is also associated with production of secondary mediators which are likely to contribute to the effects observed in microglia. Increased expression of genes which encode for IL-1β, TNF and other inflammatory cytokines is detected in the CNS of GFAP-IL6 [14] and GFAP-IFN mice (Phillip West, unpublished data). Although these caveats must be considered when interpreting the effects of these cytokines, the cytokine environments induced by IL-6 versus IFN-α are distinct. Accordingly, while GFAP-IL6, GFAP-IFN and other transgenic lines including GFAP-TNF mice exhibit clinical and molecular phenotypes with certain overlapping features, the differences are more prominent and can be attributed to the unique actions of each cytokine [160, 161]. Furthermore, in this study we observed stark differences in the signaling pathways activated by IL-6 versus IFN-α, as well as divergent alterations in the microglia molecular and cellular phenotype. However, in order to attribute the microglia responses to the direct actions of IL-6 versus IFN-α, loss-of-function experiments are required and are the focus of an ongoing study. Finally, although we did not determine the actual cytokine levels in the CNS of GFAP-IL6 and GFAP-IFN mice, the levels of cerebellar mRNA for transgenic IL-6 versus IFN-α were comparable (Phillip West, unpublished data).

The pharmacological ablation of microglia is an emerging strategy used to discern the functional significance of these cells in health and disease [2]. Accordingly, microglia ablation has enabled researchers to elucidate important actions of microglia, including maintenance of circadian rhythm [162], regulation of satiety and metabolism [163], and restraint of neuronal hyperexcitability [164]. In addition, the ablation of microglia is protective in certain neuropathological contexts but is harmful in others [165, 166], suggesting diverse functions of these cells in disease. These findings highlight the value of depleting microglia to dissect their functional significance in GFAP-IL6 and GFAP-IFN mice and this has been the approach of a further study (manuscript in preparation).

Furthermore, the production of GFAP by astrocytes is highly variable and is universally regulated in response to perturbation of the CNS [167]. Astrogliosis is readily apparent in the brain of GFAP-IL6 and GFAP-IFN mice and these cells produce increased levels of GFAP [8, 28]. Since the reactivity of astrocytes is potentially associated with the differential regulation of cytokine production in GFAP-IL6 and GFAP-IFN mice, the activation of astrocytes should also be considered when interpreting the functional state of microglia in GFAP-IL6 and GFAP-IFN mice. Detailed analysis of the molecular and cellular phenotype of astrocytes in these animals is warranted in the future and may potentially provide insight into their interaction with microglia, as well as their regulation of the microglia phenotype, during disease progression.

## Conclusions

Taken together, we demonstrate here that microglia are a major target and effector cell of IL-6 and IFN-α in vivo and respond to chronic production of these cytokines in the brain by making wide-ranging cellular, molecular and transcriptional adaptations. Importantly, microglia showed stimulus-specific responses, giving rise to unique and distinct microglia phenotypes. These findings further highlight the exquisite responsiveness of microglia to altered cytokine signaling and pathology in the CNS. In addition, the identified IL-6- and IFN-response genes here are also substantial components of the transcriptional response of microglia reported in a wide range of neuroinflammatory and neurodegenerative disorders, indicating that both cytokines likely contribute to the phenotype and function of microglia in these diseases. The stimulus-specific responses of microglia reflect known functional and pathological roles of IL-6 versus IFN-α in the brain and likely impart these cells with their unique functions in IL-6 versus IFN-α-mediated neurological disease.

## Supplementary Information

**Additional file 1.** Supplementary figures 1–11.

**Additional file 2. Table S1**. Differential gene expression analysis and top 100 enriched biological processes (BPs) of microglia from WT, GFAP-IL6 and GFAP-IFN mice from Fig. 3.

**Additional file 3. Table S2** Reads (FPKM) for common, IL-6- and IFN-α-skewed genes and top 100 enriched BPs by upregulated genes from Fig. 4.

**Additional file 4. Table S3.** Reads (FPKM) and log2 fold-changes from meta-analysis and the top 20 enriched BPs by gene clusters of interest in Fig. 7 and Fig. S8.

## Data Availability

The authors declare that all data supporting the findings of this study are available within the article and its Additional Information files. The raw RNA-seq data have been deposited in the European Nucleotide Archive under the entry code PRJEB46781.

## Abbreviations

AD: Alzheimer’s disease
ANOVA: Analysis of variance
BPs: Biological processes
BrdU: 5-Bromo-2’-deoxyuridine
CNS: Central nervous system
DEGs: Differentially expressed genes
EAE: Experimental autoimmune encephalomyelitis
eGFP: Enhanced green fluorescent protein
FACS: Fluorescence-activated cell sorting
FDR: False discovery rate
FlowSOM: Flow Self-Organizing Maps
FPKM: Fragments per kilobase of transcript per million mapped reads
GFAP: Glial fibrillary acidic protein
GO: Gene ontology
IFN: Interferon
IL: Interleukin
LPS: Lipopolysaccharide
MS: Multiple sclerosis
NMOSD: Neuromyelitis optica spectrum disorder
PCA: Principal component analysis
pS: Phospho-serine
pY: Phospho-tyrosine
STAT: Signal transducer and activator of transcription
TUNEL: Terminal deoxynucleotidyl transferase dUTP nick end labeling
UMAP: Uniform Manifold Approximation and Projection
WT: Wild type
