# Supplementary material for: The cytokines interleukin-6 and interferon-α induce distinct microglia phenotypes
Source: J Neuroinflammation. 2022 Apr 16;19:96. doi: 10.1186/s12974-022-02441-x (PMC9013466; doi:10.1186/s12974-022-02441-x)
Supplement: Supplementary file 1 — Additional file 1. Supplementary figures 1–11. [file 12974_2022_2441_MOESM1_ESM.pdf]

The cytokines interleukin-6 and interferon-α induce distinct microglia phenotypes

Phillip K. West[1], Andrew N. McCorkindale[2], Boris Guennewig[3], Thomas M. Ashhurst[2,4], Barney Viengkhou[1], Emina Hayashida[1], So Ri Jung[1], Oleg Butovsky[5], Iain L. Campbell[1] and Markus J. Hofer[1]

List of additional materials:

Fig. S1 IL-6 and IFN-α-specific signaling pathways are activated in microglia in the brain of GFAP-IL6 versus GFAP-IFN mice

Fig. S2 Source data for immunoblots in Fig. S1

Fig. S3 Microglia exhibit a more pronounced and more extensive response to IFN-α versus IL-6

Fig. S4 Expression of core response, IL-6- and IFN-α-skewed genes by microglia at 1-, 3- and 6-months of age in GFAP-IL6 and GFAP-IFN mice as measured by RTPCR, for the most part, validates the findings from RNA-seq

Fig. S5 UMAP plots showing expression levels of indicated markers on microglia and infiltrate populations from the Spectre discover workflow analysis in Fig. 5

Fig. S6 The global leukocyte landscape differs in the brain of GFAP-IL6 versus GFAP-IFN mice

Fig. S7 Gating strategy for analysis of microglia surface marker expression in Fig. 6

Fig. S8 Genes from microglia gene expression datasets that are differentially expressed in at least 4 different conditions organize into 22 co-regulated clusters by hierarchical clustering

Fig. S9 Meta-analysis of microglia gene expression datasets reveals co-regulated gene clusters associated with universal damage response and response to chronic stimuli

Fig. S10 Microglia-specific and cytokine-regulated genes contribute to the universal danger and chronic response gene clusters

Fig. S11 Meta-analysis of microglia gene expression datasets in an array of neuropathological states reveals co-regulated gene clusters associated with IL-6- and IFN-induced responses

Table S1 Differential gene expression analysis and top 100 enriched biological processes (BPs) of microglia from WT, GFAP-IL6 and GFAP-IFN mice from Fig. 3

Table S2 Reads (FPKM) for common, IL-6- and IFN-α-skewed genes and top 100 enriched BPs by upregulated genes from Fig. 4

Table S3 Reads (FPKM) and $\log_2$ fold-changes from meta-analysis and the top 20 enriched BPs by gene clusters of interest in Fig. 7 and Fig. S8

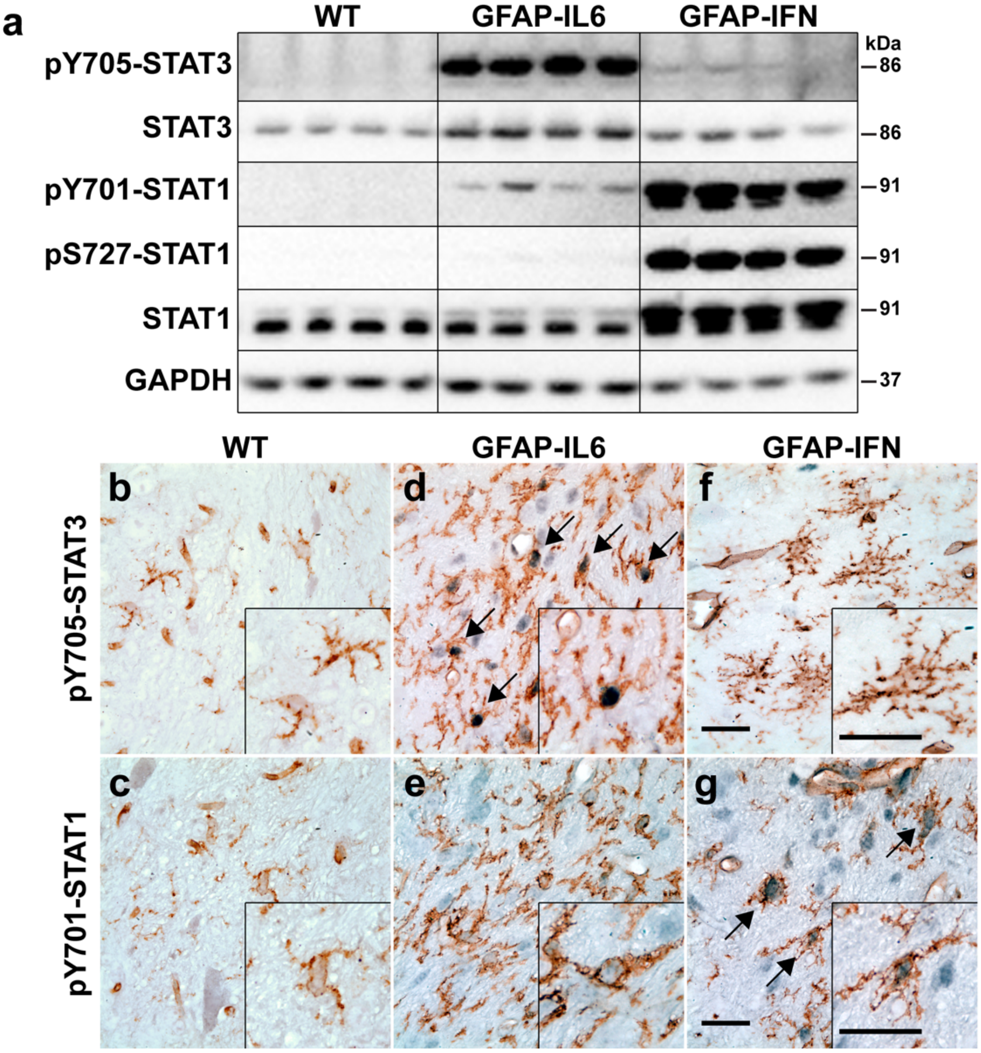

Fig. S1 IL-6 and IFN-α-specific signaling pathways are activated in microglia in the brain of GFAP-IL6 versus GFAP-IFN mice. **a** Western blot analysis of cerebellar protein lysates (30 μg) from 1-month-old WT, GFAP-IL6 and GFAP-IFN mice. n=4 mice/genotype. **b-g** Immunohistochemistry for pY705-STAT3 **(b, d, f)** or pY701-STAT1 **(c, e, g)** (dark purple), combined with histochemistry for lectin (brown), was performed on brain sections from 1-month-old WT **(b, c)**, GFAP-IL6 **(d, e)** and GFAP-IFN **(f, g)** mice. Scale bars, 25 μm. Representative images from the cerebellum are shown. Arrows indicate lectin-positive microglia positive for nuclear pY705-STAT3 or pY701-STAT1

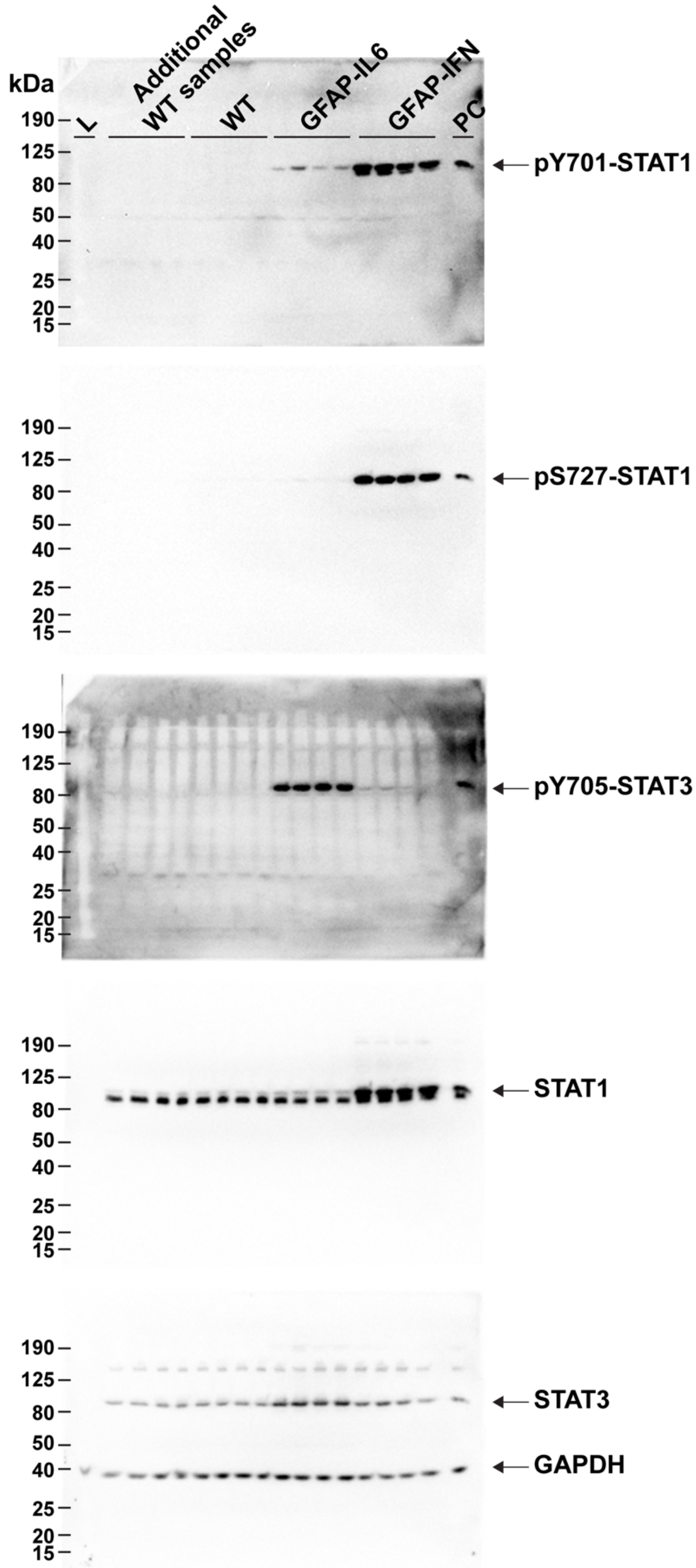

Fig. S2 Source data for immunoblots in Fig. S1. L = ladder, PC = positive control

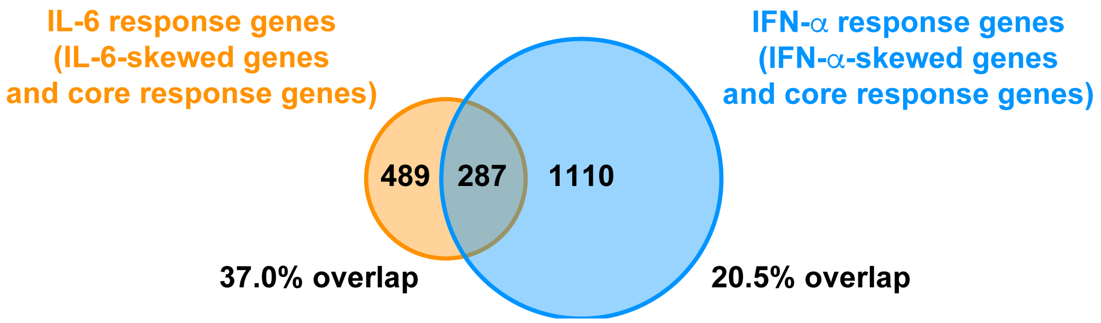

Fig. S3 Microglia exhibit a more pronounced and more extensive response to IFN-α versus IL-6. Venn diagram in which IL-6 response genes (including both IL-6-skewed genes and core response genes) are compared with IFN-α response genes (including both IFN-α-skewed genes and core response genes)

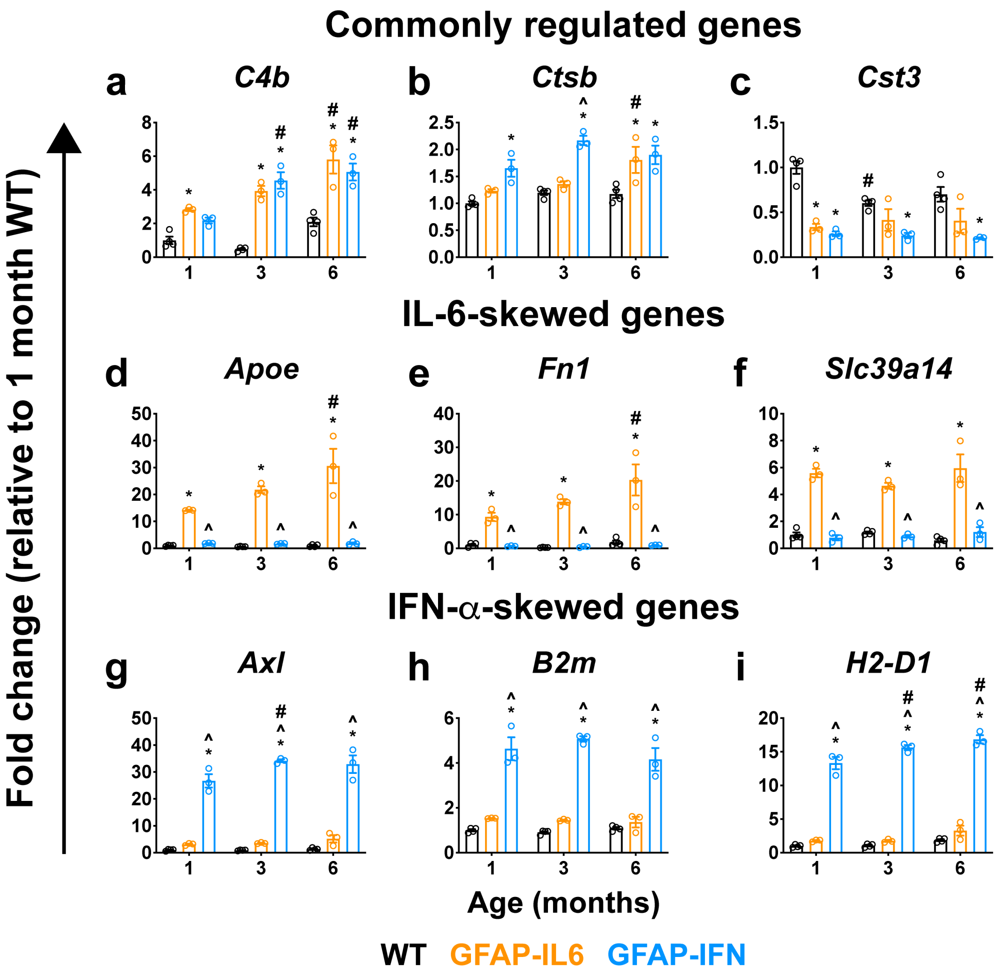

Fig. S4 Expression of core response, IL-6- and IFN-α-skewed genes by microglia at 1-, 3- and 6-months of age in GFAP-IL6 and GFAP-IFN mice as measured by RTPCR, for the most part, validates the findings from RNA-seq. **a-i** Gene expression of **(a)** *C4b*, **(b)** *Ctsb*, **(c)** *Cst3*, **(d)** *Apoe*, **(e)** *Fn1*, **(f)** *Slc39a14*, **(g)** *Axl*, **(h)** *B2m* and **(i)** *H2-D1* in microglia from the brain of 1-, 3- and 6-month-old WT, GFAP-IL6 and GFAP-IFN mice detected by RTPCR. n=3-4 mice/group. Graphs show individual values per mouse and mean ± SEM. *, $p<0.05$ compared with WT of the same age; ^, $p<0.05$ compared with GFAP-IL6 of the same age; #, $p<0.05$ compared with 1-month-old of the same genotype using two-way ANOVA with Tukey's post-test

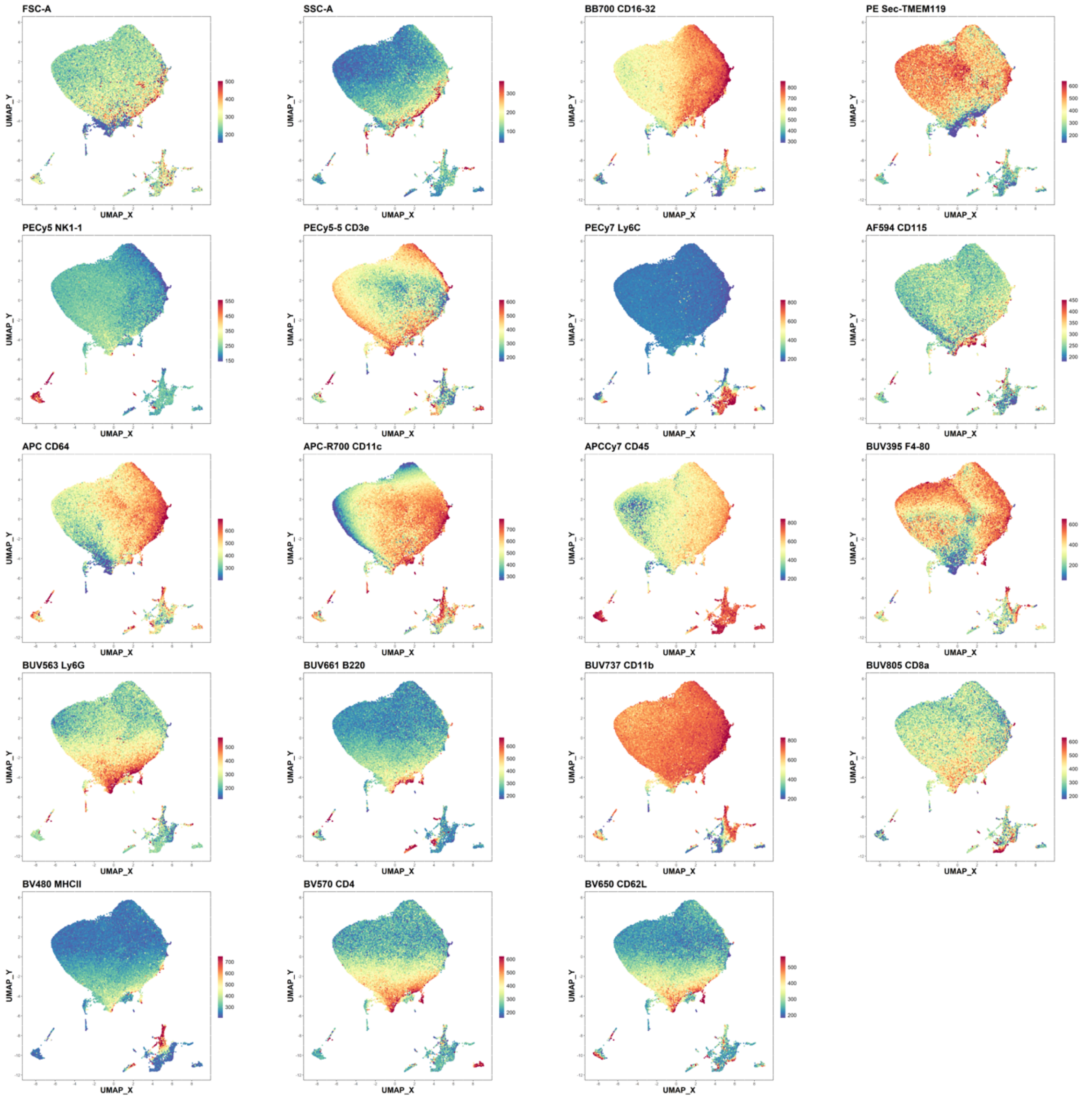

Fig. S5 UMAP plots showing expression levels of indicated markers on microglia and infiltrate populations from the Spectre discover workflow analysis in Fig. 5. Leukocytes were isolated, stained with fluorophore-conjugated antibodies and were analyzed by flow cytometry, followed by the Spectre package. Each UMAP plot shows the relative expression of each cellular parameter, from low (blue) to high (red)

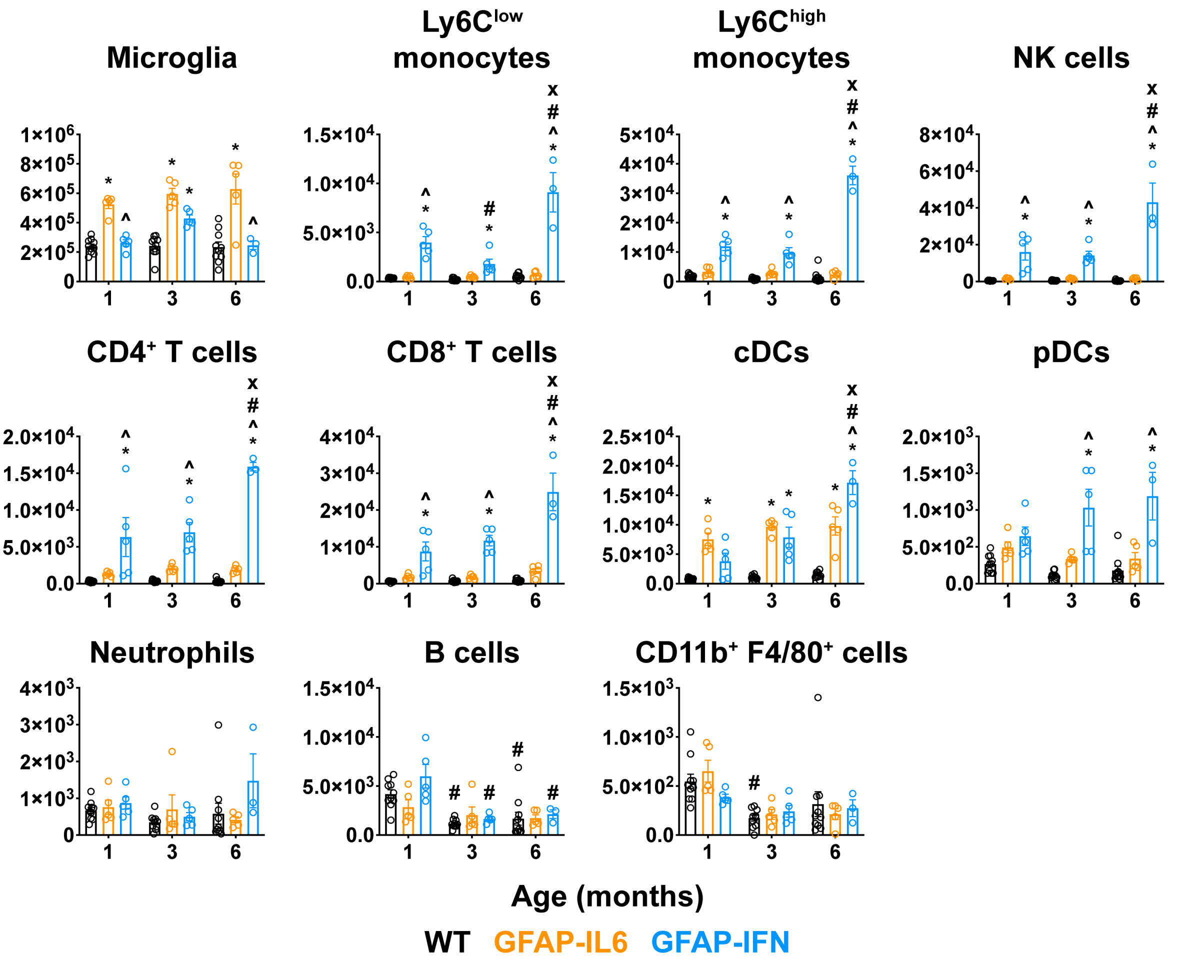

Fig. S6 The global leukocyte landscape differs in the brain of GFAP-IL6 versus GFAP-IFN mice. Numbers of microglia and leukocyte subpopulations in the whole brain of 1-, 3- and 6-month-old WT, GFAP-IL6 and GFAP-IFN mice. n=3-5 mice/group. Graphs show individual values per mouse and mean ± SEM. *, $p<0.05$ compared with WT; ^, $p<0.05$ compared with GFAP-IL6; #, $p<0.05$ compared with 1-month-old of same genotype; x, $p<0.05$ compared with 3-month-old of same genotype using two-way ANOVA with Tukey's post-test

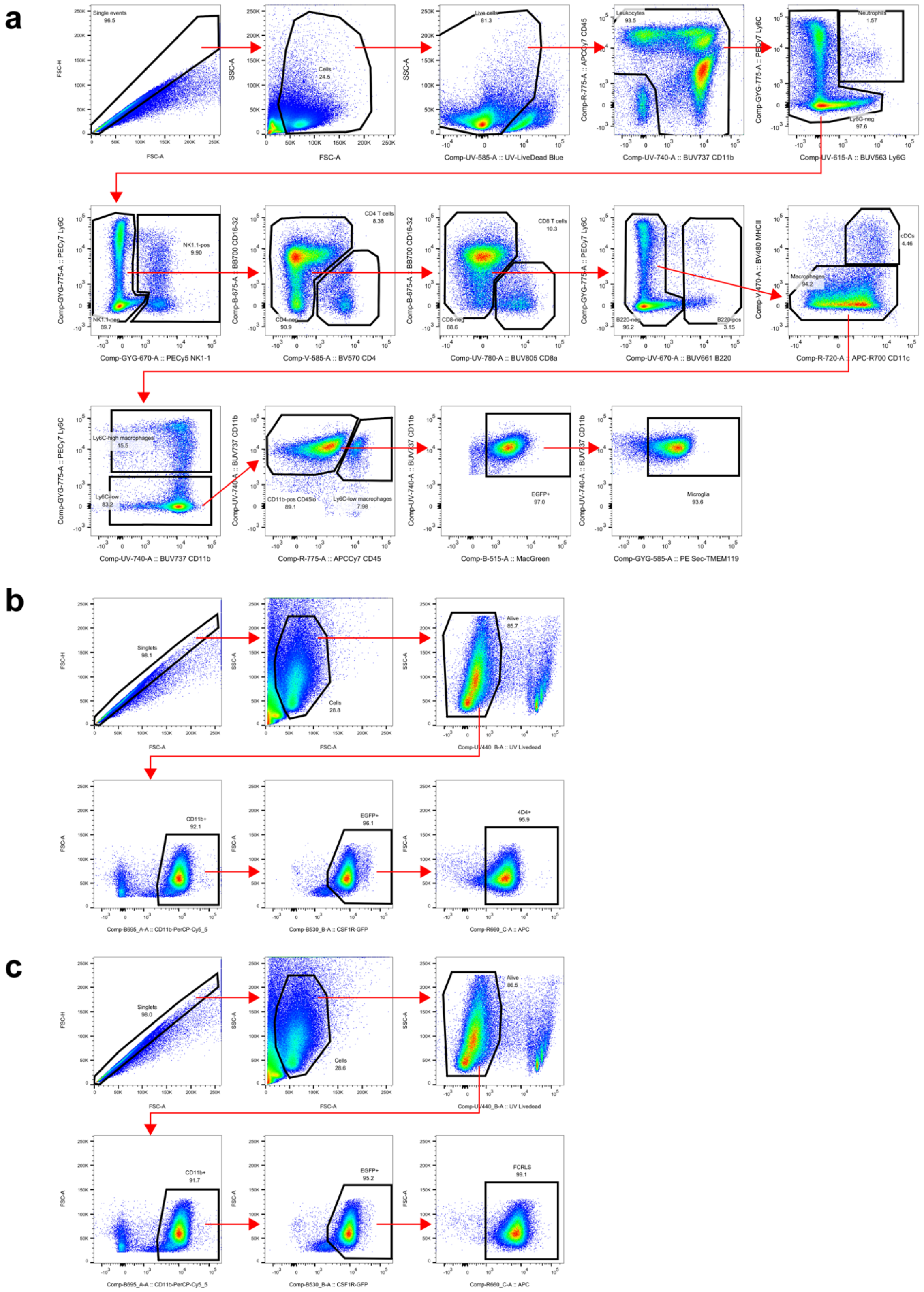

Fig. S7 Gating strategy for analysis of microglia surface marker expression in Fig. 6. **a** Microglia were gated as UV-Live/Dead^{-} Ly6G^{-} NK1.1^{-} CD4^{-} CD8^{-} B220^{-} MHC-II^{low} Ly6C^{low} CD11b^{+} CD45^{low} eGFP^{+} TMEM119^{+} cells. Gates were placed based on unstained controls and

fluorescence minus one (FMO) controls. From the microglia gate, MFI was quantified for TMEM119, CD16/32, CD64, CD11b and SCA-1 and the percentage of microglia positive for CD64, CD11c, SCA-1, MHC-II, CD80 and CD86 were determined as shown in Fig. 6. **b-c** Microglia were gated as UV-Live/Dead^{-} CD11b^{+} eGFP^{+} 4D4^{+} **(b)** or UV-Live/Dead^{-} CD11b^{+} eGFP^{+} FCRLS^{+} **(c)**. Gates were placed based on unstained controls and FMOs. From the microglia gate, MFI was quantified for 4D4, FCRLS and MHC-I and the percentage of microglia positive for MHC-I were determined as shown in Fig. 6

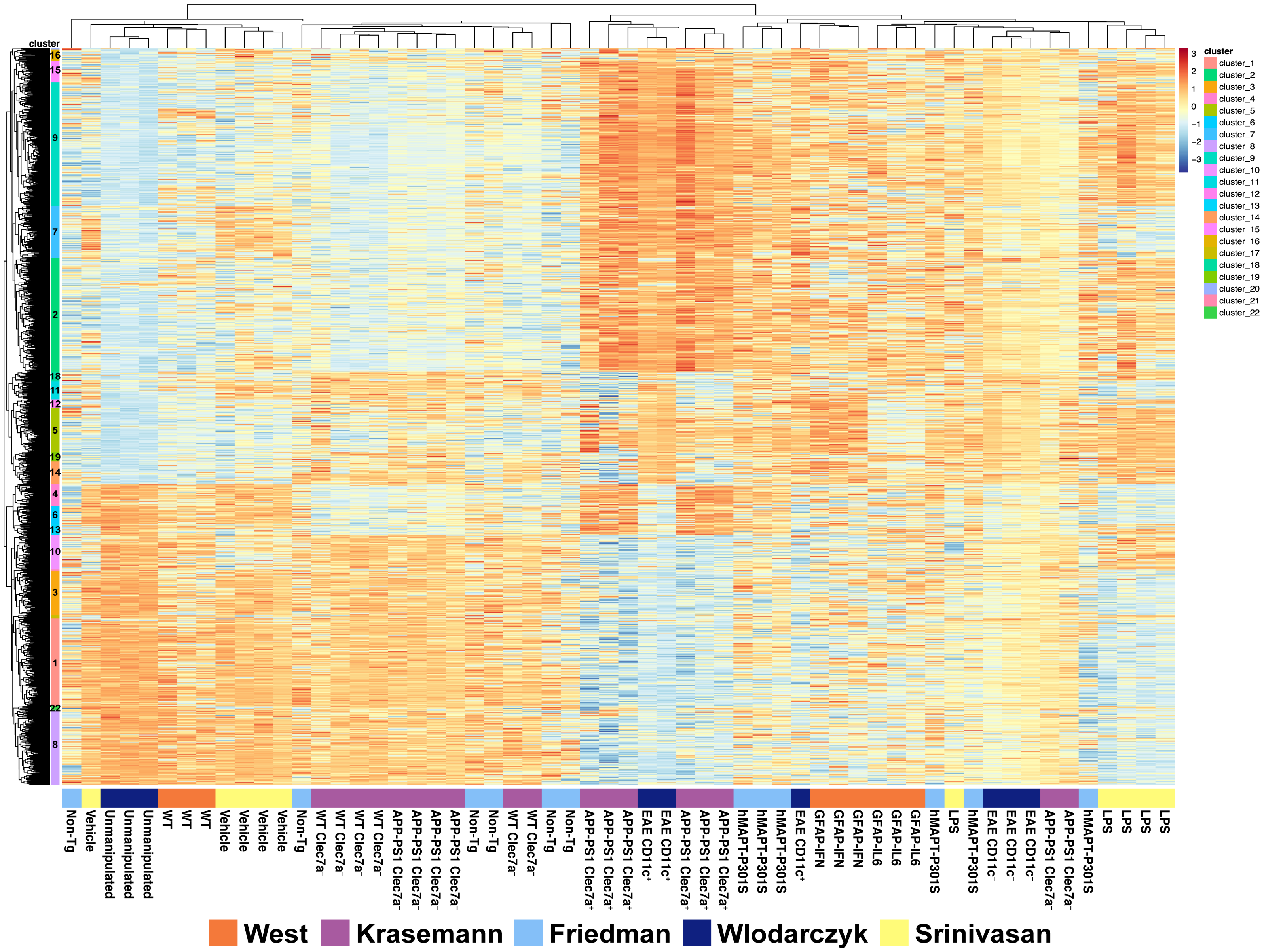
cluster
16
15
9
7
2
18
11
12
5
19
14
4
6
13
10
3
1
22
8
Non-Tg
Vehicle
Unmanipulated
Unmanipulated
Unmanipulated
WT
WT
WT
Vehicle
Vehicle
Vehicle
Vehicle
Non-Tg
WT Clec7a-
WT Clec7a-
WT Clec7a-
WT Clec7a-
APP-PS1 Clec7a-
APP-PS1 Clec7a-
APP-PS1 Clec7a-
APP-PS1 Clec7a-
Non-Tg
Non-Tg
WT Clec7a-
WT Clec7a-
Non-Tg
Non-Tg
APP-PS1 Clec7a+
APP-PS1 Clec7a+
APP-PS1 Clec7a+
EAE CD11c+
EAE CD11c+
APP-PS1 Clec7a+
APP-PS1 Clec7a+
APP-PS1 Clec7a+
hMAPT-P301S
hMAPT-P301S
hMAPT-P301S
EAE CD11c+
GFAP-IFN
GFAP-IFN
GFAP-IFN
GFAP-IL6
GFAP-IL6
GFAP-IL6
hMAPT-P301S
LPS
hMAPT-P301S
EAE CD11c-
EAE CD11c-
EAE CD11c-
APP-PS1 Clec7a-
APP-PS1 Clec7a-
hMAPT-P301S
LPS
LPS
LPS
LPS
3
2
1
0
−1
−2
−3
cluster
cluster_1
cluster_2
cluster_3
cluster_4
cluster_5
cluster_6
cluster_7
cluster_8
cluster_9
cluster_10
cluster_11
cluster_12
cluster_13
cluster_14
cluster_15
cluster_16
cluster_17
cluster_18
cluster_19
cluster_20
cluster_21
cluster_22
West
Krasemann
Friedman
Wlodarczyk
Srinivasan

Fig. S8 Genes from microglia gene expression datasets that are differentially expressed in at least 4 different conditions organize into 22 co-regulated clusters by hierarchical clustering

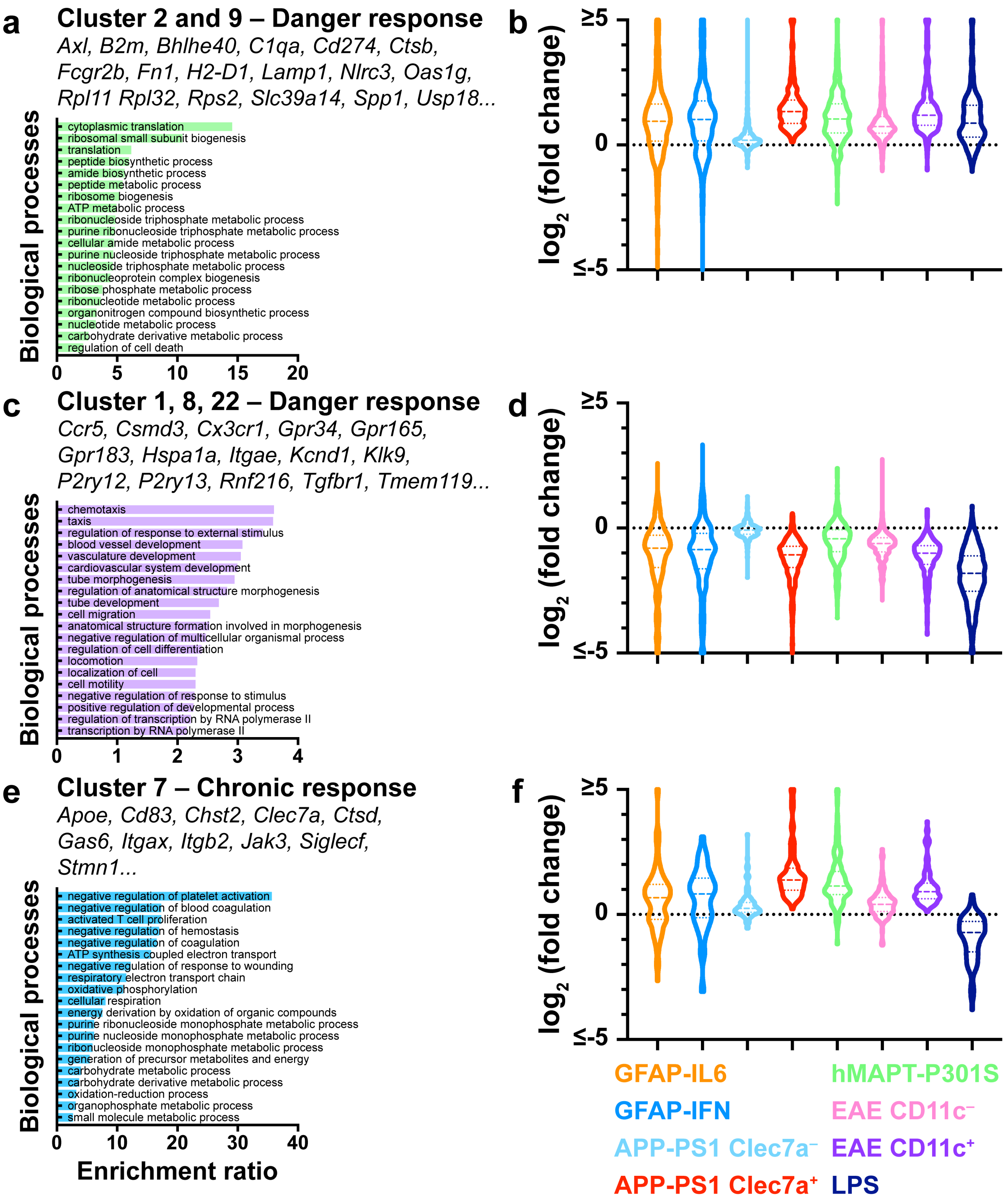

Fig. S9 Meta-analysis of microglia gene expression datasets reveals co-regulated gene clusters associated with universal damage response and response to chronic stimuli. **a** GO analysis of the biological processes of upregulated universal danger response genes (cluster 2 and 9). **b** Differential expression of cluster 2 and 9 genes by microglia responding to different neuropathological stimuli. **c** GO analysis of the biological processes of downregulated universal danger response genes (cluster 1, 8 and 22). **d** Differential expression of cluster 1, 8

and 22 genes by microglia responding to different neuropathological stimuli. **e** GO analysis of the biological processes of the chronic response genes (cluster 7). **f** Differential expression of cluster 7 genes by microglia responding to different neuropathological stimuli. For **(a, c, e)**, significantly enriched GOs (FDR<0.05) are shown. For **(b, d, f)**, differential expression was calculated by compared each condition with its respective control. Dashed lines represent median $\log_2$ expression and dotted lines represent quartiles

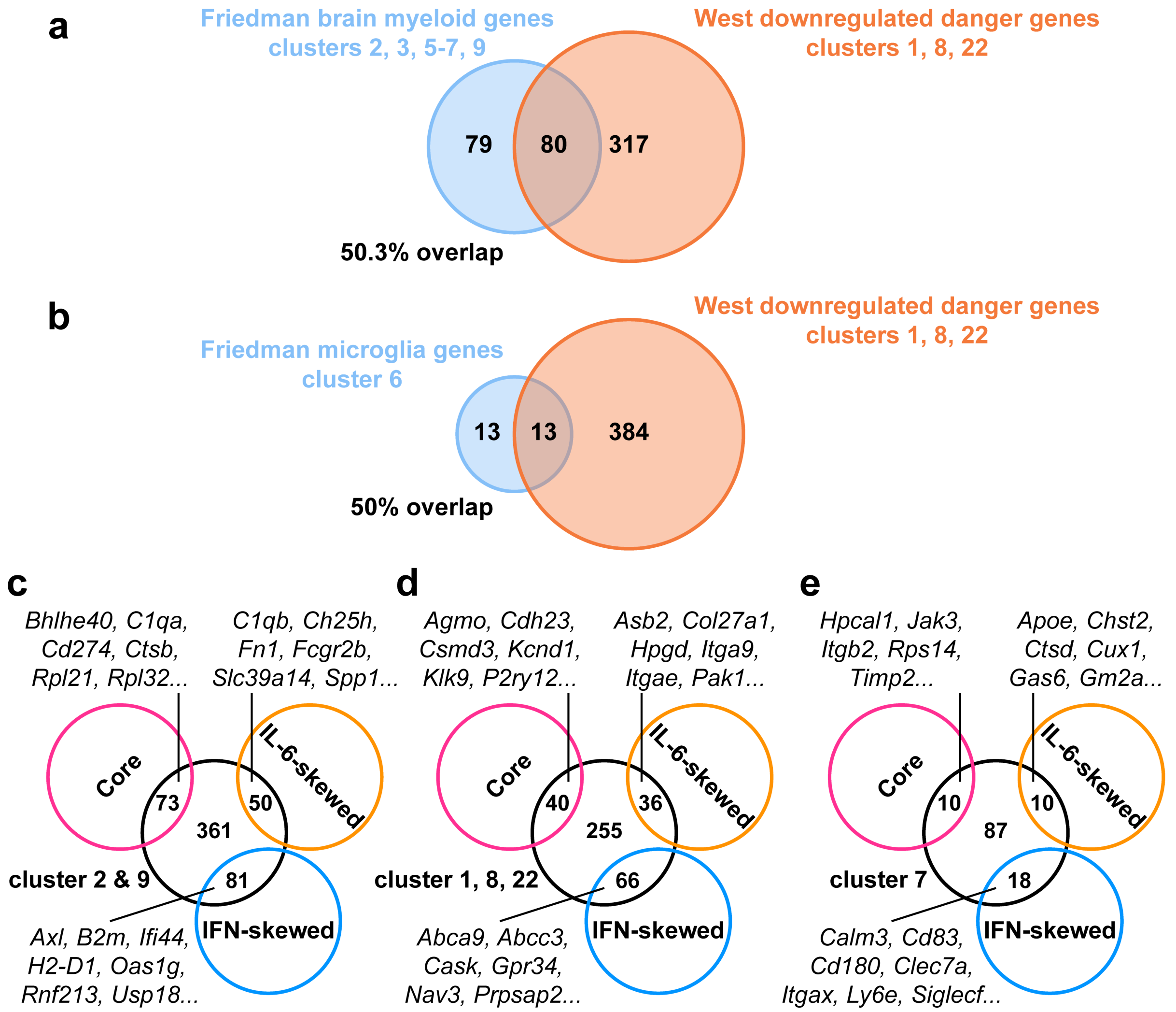

Fig. S10 Microglia-specific and cytokine-regulated genes contribute to the universal danger and chronic response gene clusters. **a** Venn diagram in which brain myeloid genes elevated in resident brain myeloid cells relative to infiltrating and peripheral macrophages (Friedman clusters 2, 3, 5-7 and 9) identified by Friedman *et al.* [1] were compared with the downregulated danger response genes (clusters 1, 8 and 22) identified in our study. **b** Venn diagram in which microglia-specific genes elevated in parenchymal microglia relative to perivascular macrophages (Friedman cluster 6) identified by Friedman *et al.* [1] were compared with the downregulated danger response genes (clusters 1, 8 and 22) identified in our study. **c-e** Venn diagrams showing the overlap between commonly regulated, IL-6- and IFN-α-skewed genes and genes in cluster 2 and 9 **(c)**, 1, 8 and 22 **(d)** and 7 **(e)**. For **(c, e)**, clusters were compared

with upregulated core, IL-6- and IFN-α-skewed genes. For **(d)**, clusters were compared with downregulated core, IL-6- and IFN-α-skewed genes

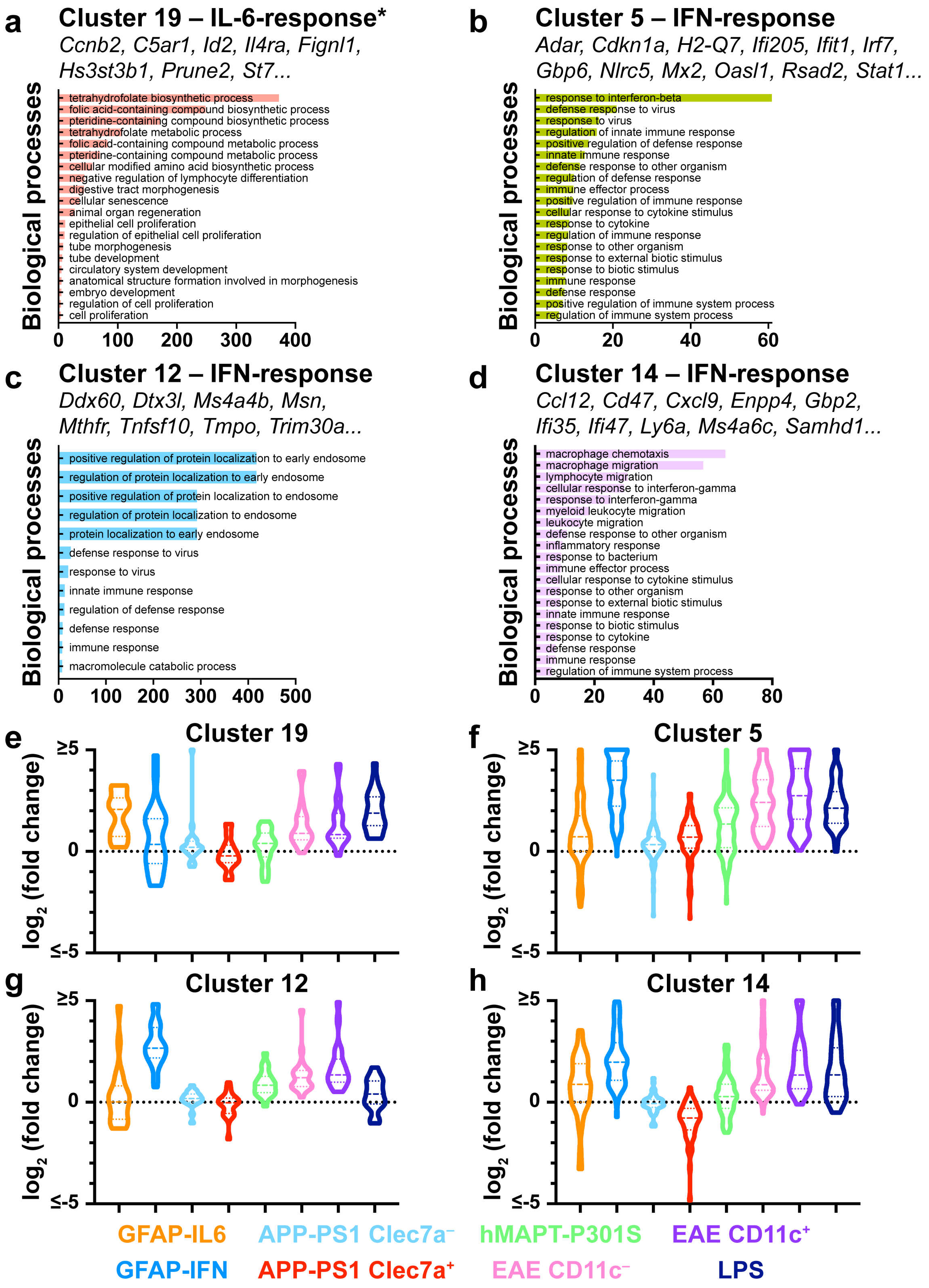

Fig. S11 Meta-analysis of microglia gene expression datasets in an array of neuropathological states reveals co-regulated gene clusters associated with IL-6- and IFN-induced responses. **a-d** GO analysis of the biological processes of IL-6-response genes (cluster 19) **(a)** and IFN-

response genes from clusters 5 **(b)**, 12 **(c)** and 14 **(d)**. **e-h** Differential expression of cluster 19 **(e)**, 5 **(f)**, 12 **(g)** and 14 **(h)** genes by microglia in response to distinct neuropathological stimuli. For **(b-d)**, significantly enriched GOs (FDR<0.05) are shown. For **(e-h)**, differential expression was calculated by comparing each condition with its respective control. Dashed lines represent median $\log_2$ expression and dotted lines represent quartiles. *N.B. The FDRs for the enriched biological processes in cluster 19 were >0.05 and therefore not statistically significant

References

1. Friedman BA, Srinivasan K, Ayalon G, Meilandt WJ, Lin H, Huntley MA, et al. Diverse brain myeloid expression profiles reveal distinct microglial activation states and aspects of Alzheimer's disease not evident in mouse models. Cell Rep. 2018;22(3):832-47.
